# Supplementary material for: Under‐Oil Autonomously Regulated Oxygen Microenvironments: A Goldilocks Principle‐Based Approach for Microscale Cell Culture
Source: Adv Sci (Weinh). 2022 Feb 4;9(10):2104510. doi: 10.1002/advs.202104510 (PMC8981459; doi:10.1002/advs.202104510)
Supplement: Supplementary file 1 — Supporting Information [file ADVS-9-2104510-s001.pdf]

ADVANCED SCIENCE
Open Access

Supporting Information

for *Adv. Sci.*, DOI 10.1002/advs.202104510

Under-Oil Autonomously Regulated Oxygen Microenvironments: A Goldilocks Principle-Based Approach for Microscale Cell Culture

Chao Li, Mouhita Humayun, Glenn M. Walker, Keon Young Park, Bryce Connors, Jun Feng, Molly C. Pellitteri Hahn, Cameron O. Scarlett, Jiayi Li, Yanbo Feng, Ryan L. Clark, Hunter Hefti, Jonathan Schrope, Ophelia S. Venturelli* and David J. Beebe**

ADVANCED SCIENCE
Open Access

Supporting Information

for *Adv. Sci.,* DOI: 10.1002/advs.202104510

Under-Oil Autonomously Regulated Oxygen Microenvironments: A Goldilocks Principle-Based Approach for Microscale Cell Culture

Chao Li, Mouhita Humayun, Glenn M. Walker, Keon Young Park, Bryce Connors, Jun Feng, Molly C. Pellitteri Hahn, Cameron O. Scarlett, Jiayi Li, Yanbo Feng, Ryan L. Clark, Hunter Hefti, Jonathan Schrope, Ophelia S. Venturelli,* and David J. Beebe**

Supplementary Information for

Under-Oil Autonomously Regulated Oxygen Microenvironments: A Goldilocks Principle-Based Approach for Microscale Cell Culture

Chao Li, Mouhita Humayun, Glenn M. Walker, Keon Young Park, Bryce Connors, Jun Feng, Molly C. Pellitteri Hahn, Cameron O. Scarlett, Jiayi Li, Yanbo Feng, Ryan L. Clark, Hunter Hefti, Jonathan Schrope, Ophelia S. Venturelli,* and David J. Beebe**

Correspondence to: djbeebe@wisc.edu; venturelli@wisc.edu; cli479@wisc.edu

This PDF file includes:

Figures S1 to S14
Tables S1 and S2
Supplementary References 1 and 2
SI Section - COMSOL Tutorial

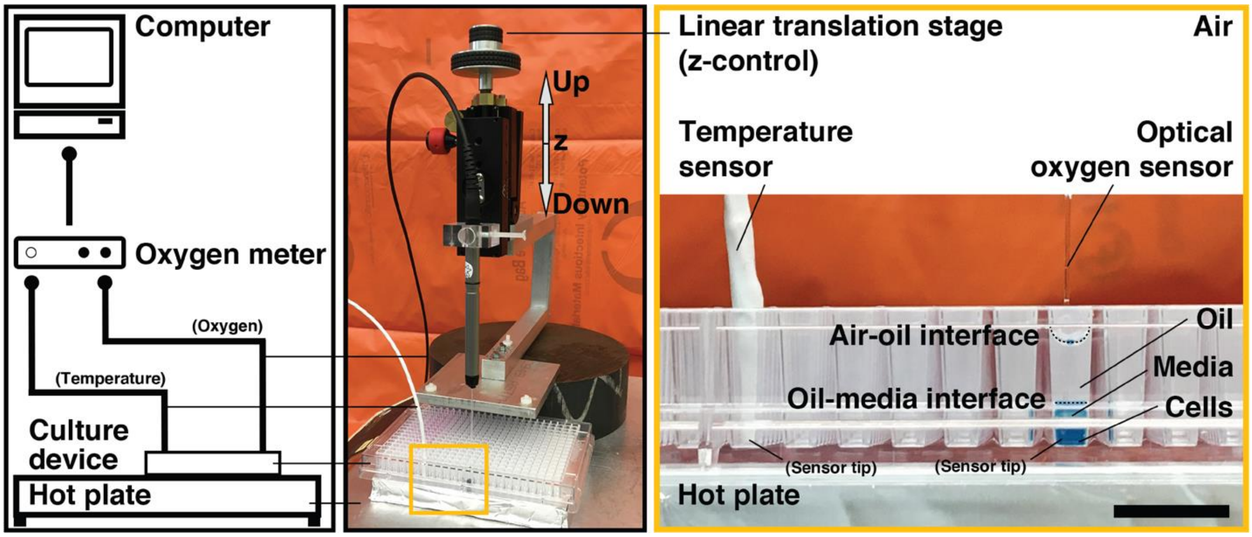

Figure S1. Camera pictures showing the setup of UOMS in air with a 384-well plate and the optical oxygen sensor system for the measurement of POC. The well contains 20 µl of media (with blue food color for visualization), overlaid with 50 µl of silicone oil (5 cSt). For a standard 384 well, 10 µl in volume leads to about 1 mm in depth. In UOMS cell culture, cells are seeded on the bottom of a well. The oxygen sensor is mounted on a linear translation stage for accurate position control in z-direction, resting on the cell layer during the measurement. The temperature sensor is submerged in a spare well filled with deionized water (50 µl) for real-time temperature compensation. Scale bar, 1 cm.

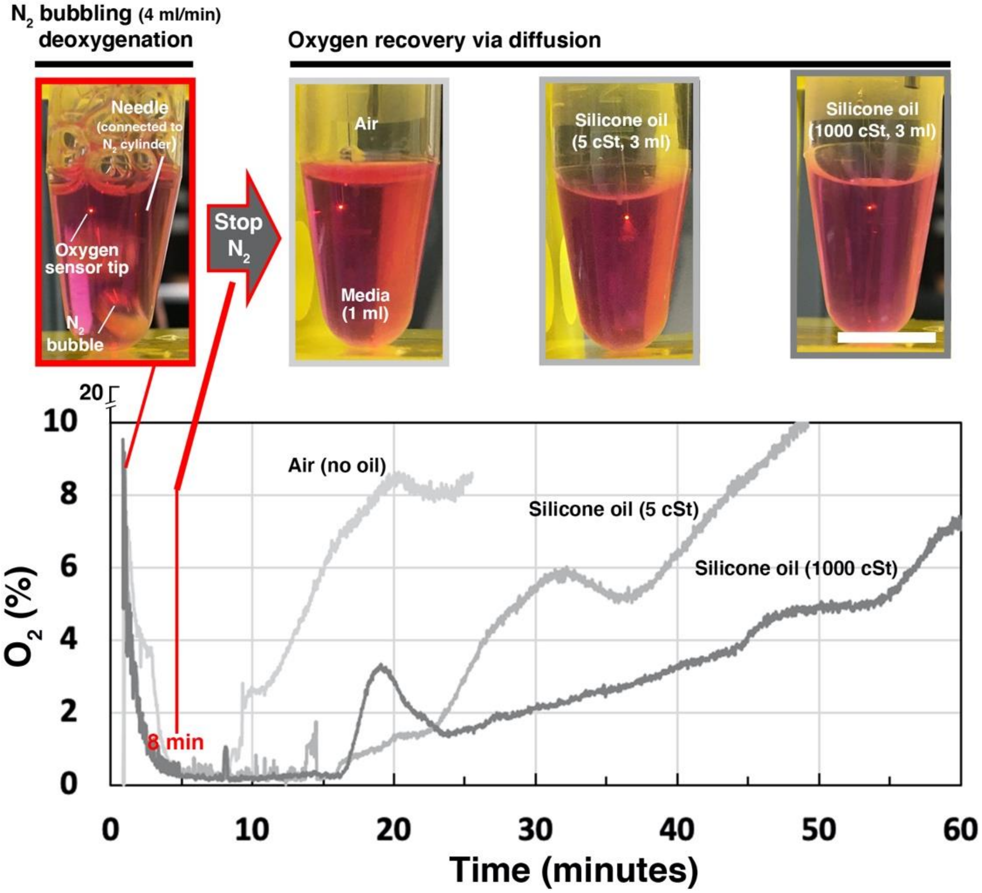

Figure S2. Oxygen diffusion test of silicone oil with different viscosities. Cell culture media (DMEM + 10% FBS) was deoxygenated to 0% O_2 by N_2 bubbling at a gas flow rate of about 4 ml/min. N_2 bubbling stopped at around 8 min. The media was overlaid with no oil (purged with air) or silicone oil in 5 cSt and 1000 cSt, respectively. The oxygen recovery process was recorded with the oxygen sensor tip kept at about 1 mm below the air/media (or oil/media) interface until it reached about 10% O_2. 3 ml of oil added on top of the media in the 5 ml centrifuge tube leads to about 18 mm in the oil depth. Note that the O_2 signal fluctuations recorded in the conditions with oil overlay were caused by the metastability and spontaneous adjustment of the oil/media meniscus during measurements. Scale bar, 10 mm.

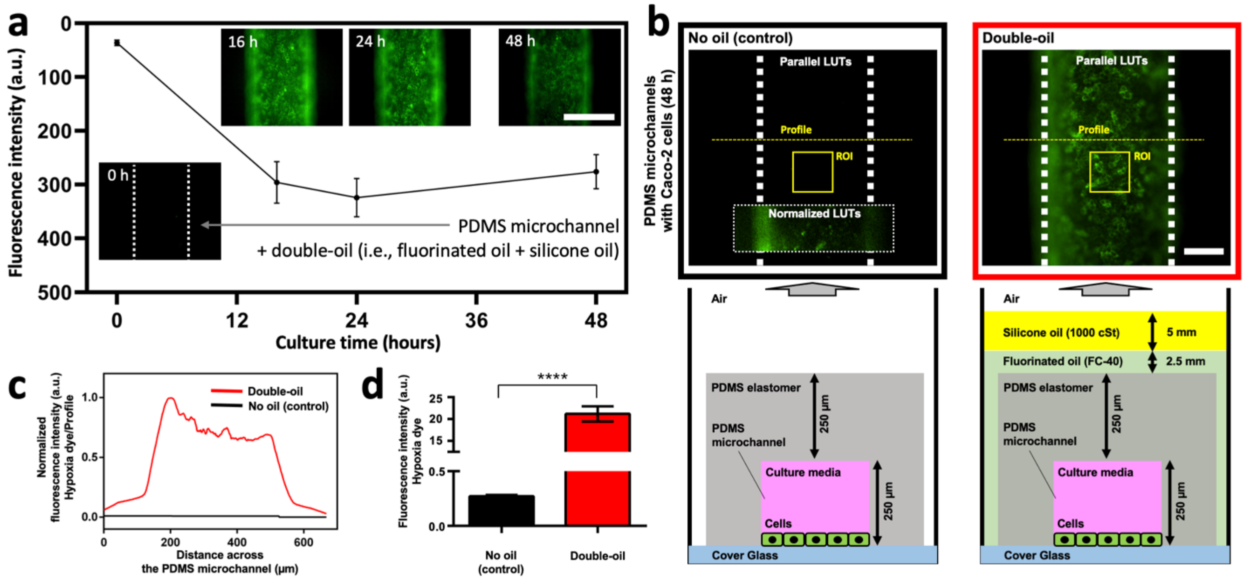

Figure S3. Oxygen diffusion in PDMS microchannels with and without oil overlay. a) The kinetics of IOC (monitored by the hypoxia dye, green) in 48 h from a PDMS microchannel with a confluent (Caco-2) cell monolayer and double-oil overlay. Scale bar, 500 µm. b) The fluorescent images of hypoxia dye from the microchannels with no oil (control, left) and double-oil [i.e., fluorinated oil (Fluorinert FC-40) + silicone oil (1000 cSt), right] cultured for 48 h. Parallel LUTs (with exposure time of 500 ms), were applied for the comparison of fluorescence intensity. [Inset of no oil (control)] A fluorescent image with normalized LUTs to visualize the cells in the microchannel. The white dashed lines indicate the boundary of the microchannels. The channel dimensions are about 2500 µm in length (not fully shown in the images), 600 µm in width, and 250 µm in height. Scale bar, 200 µm. The schematic under each microchannel shows the cross section of the microchannels perpendicular to the length direction. c) The profiles of fluorescence intensity (normalized) across the microchannels [the yellow dashed lines in (b)]. d) The bar graph of IOC (fluorescence intensity of hypoxia dye) of each microchannel. The ROIs are indicated by the yellow boxes in (b). Error bars, mean ± s.d. ****$P \leq 0.0001$.

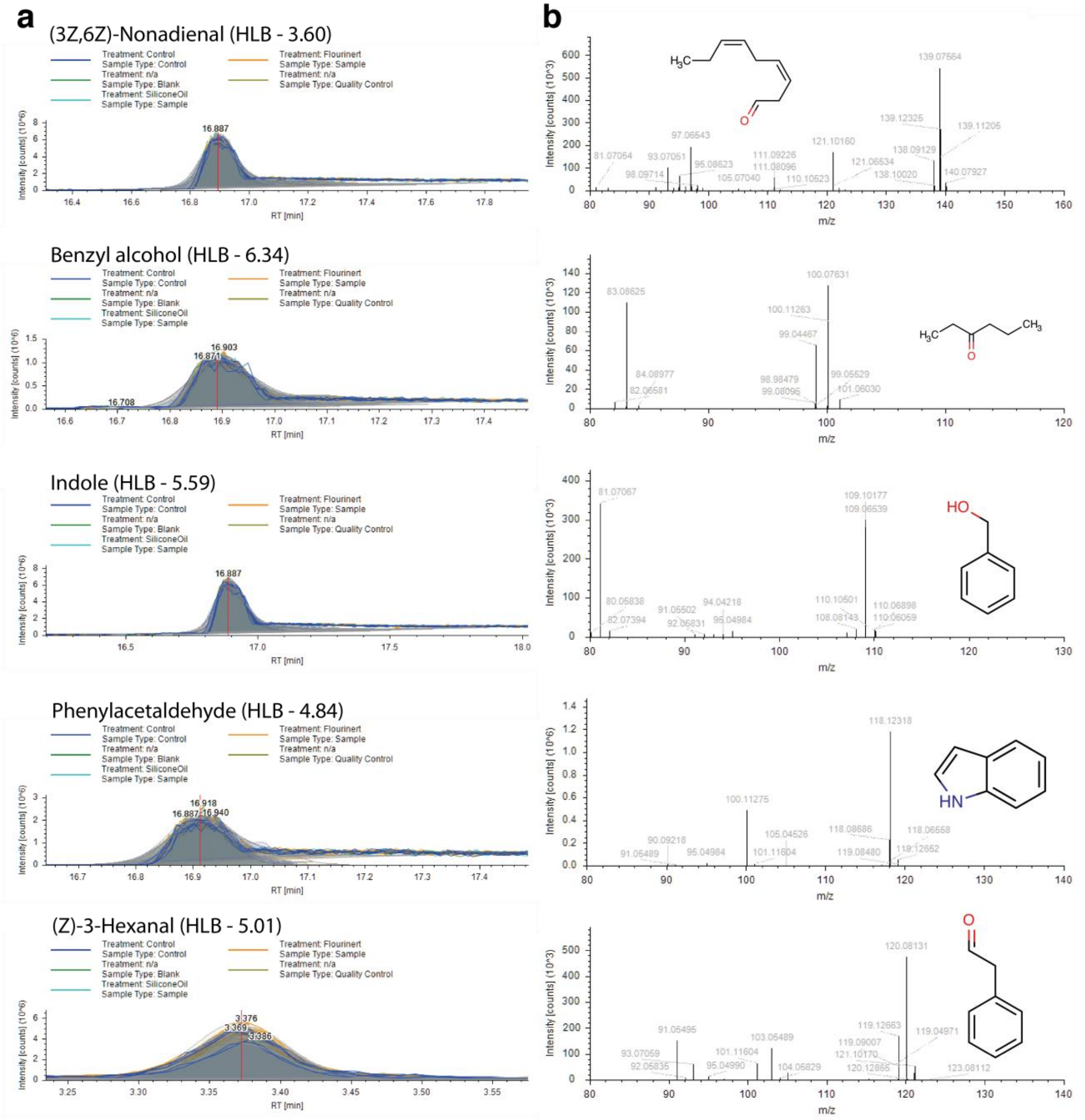

Figure S4. Raw UPLC-MS media analysis results. a) EICs (raw data, unsmoothed) of the five identified lipophilic compounds with their HLB values. Intensity of the signal at a chosen mass-to-charge (m/z) value is plotted against the retention time (RT). "Treatment: Control" is for the internal standard condition. See Methods for details of the other conditions. b) ms/ms fragments spectra of each compound.

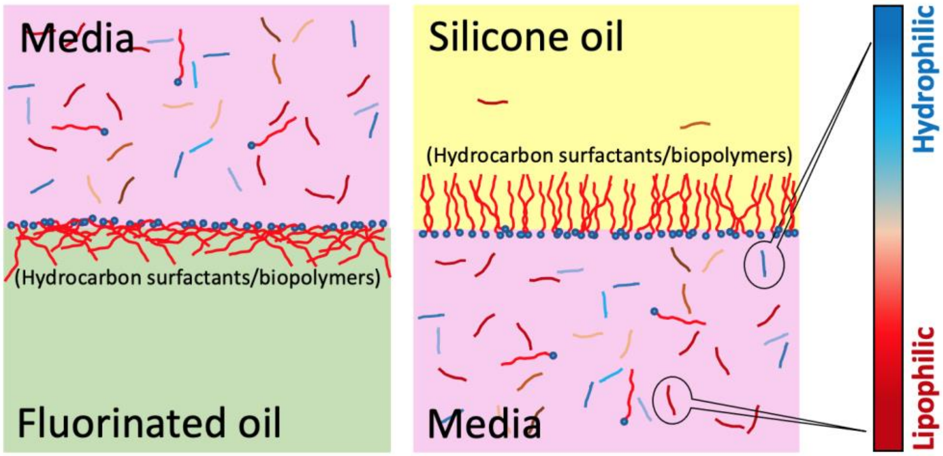

Figure S5. The proposed mechanisms for high retention of lipophilic molecules in media-fluorinated oil and media-silicone oil conditions. Studies showed that the hydrocarbon surfactants and biopolymers can get accumulated at the media-oil interfaces in a very fast rate (less than a second), which acts as a barrier that mitigates or prevents the extraction of low or nonvolatile molecules by the oil phase. In addition, the chemical inertness of fluorinated oil leads to insolubility of most of the hydrocarbon/organic molecules.

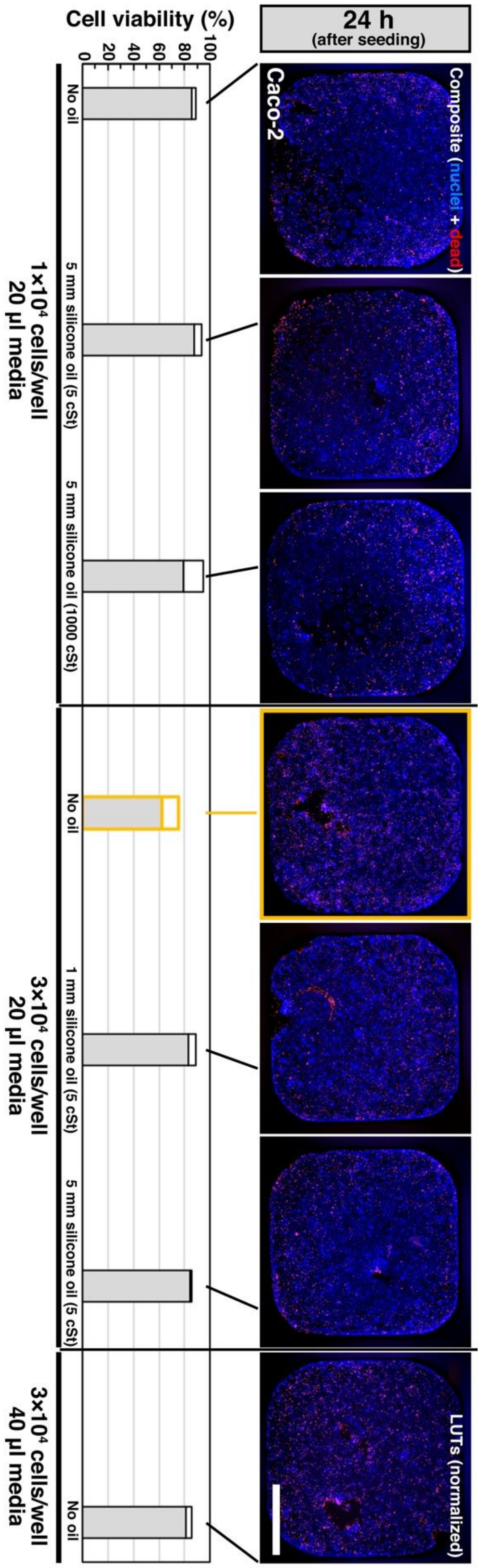

Figure S6. Cell viability of Caco-2 cultured with and without oil (silicone oil, 5 cSt) overlay (24 h after cell seeding with hypoxia dye). For a standard 384 well, 10 µl in volume leads to about 1 mm in depth. The fluorescent images were all processed with normalized LUTs for visualization. Scale bar, 1 mm.

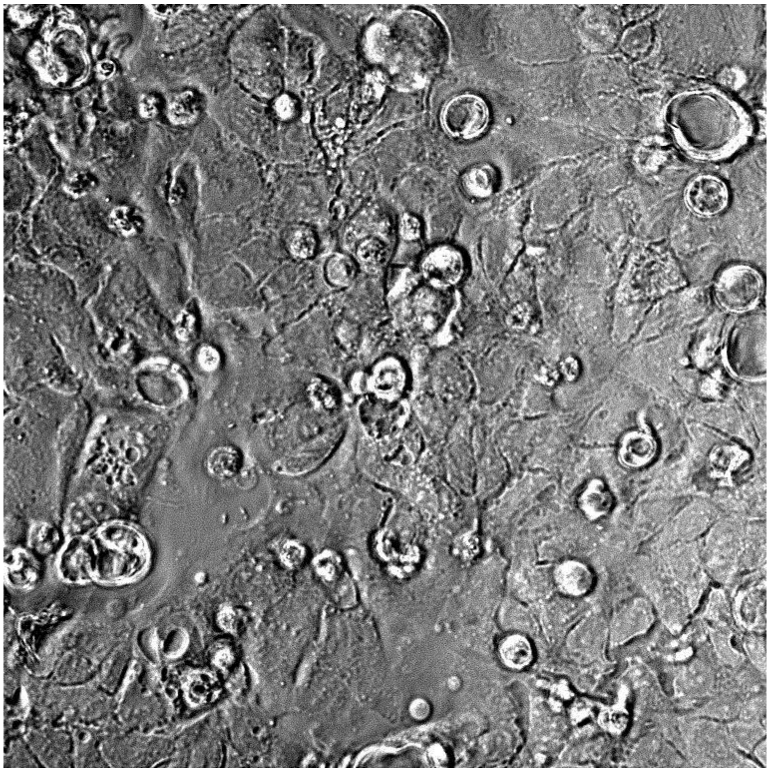

Caco-2, 1×10^4 cells/well, no oil, 24 h (bright field)

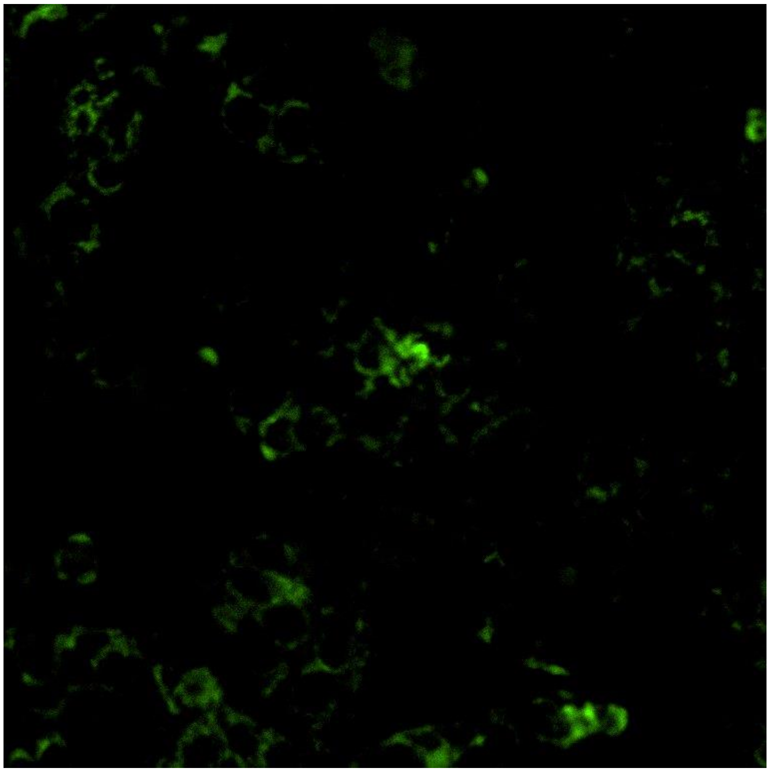

Caco-2, 1×10^4 cells/well, no oil, 24 h (hypoxia dye)

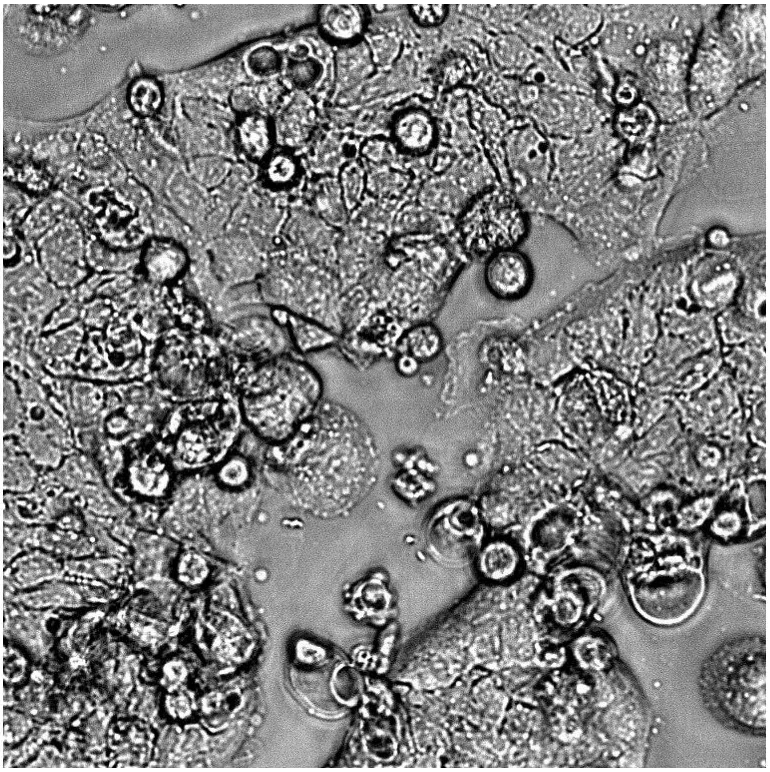

Caco-2, 1×10^4 cells/well, media-2 mm, silicone oil-5 mm-1000 cSt, 24 h (bright field)

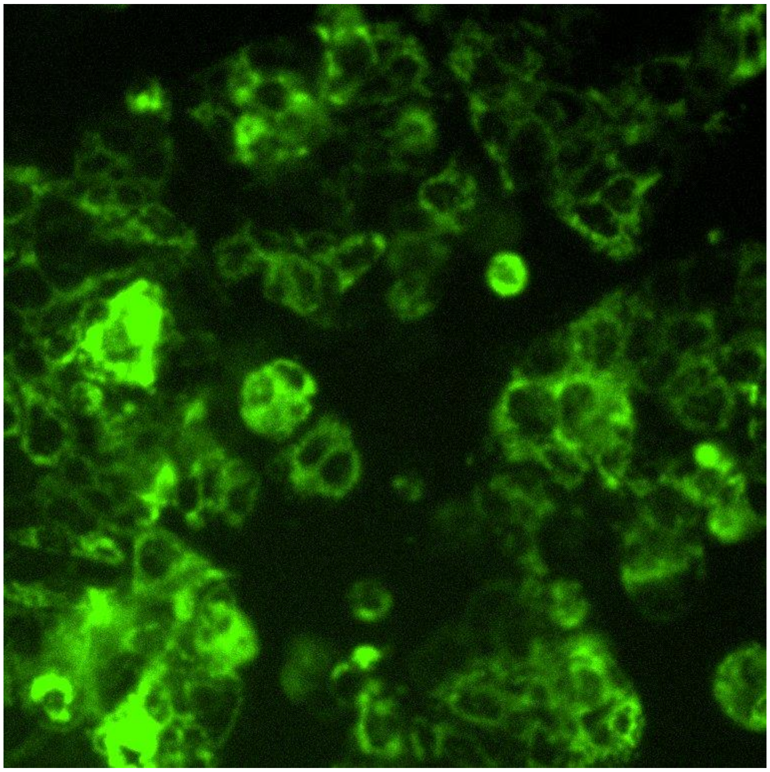

Caco-2, 1×10^4 cells/well, media-2 mm, silicone oil-5 mm-1000 cSt, 24 h (hypoxia dye)

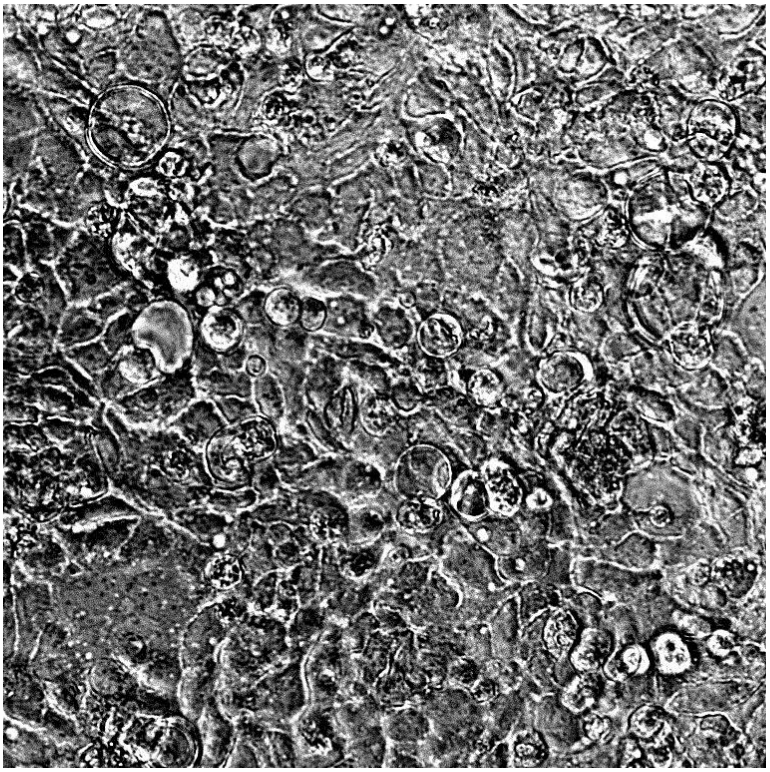

Caco-2, 3×10^4 cells/well, no oil, 24 h (bright field)

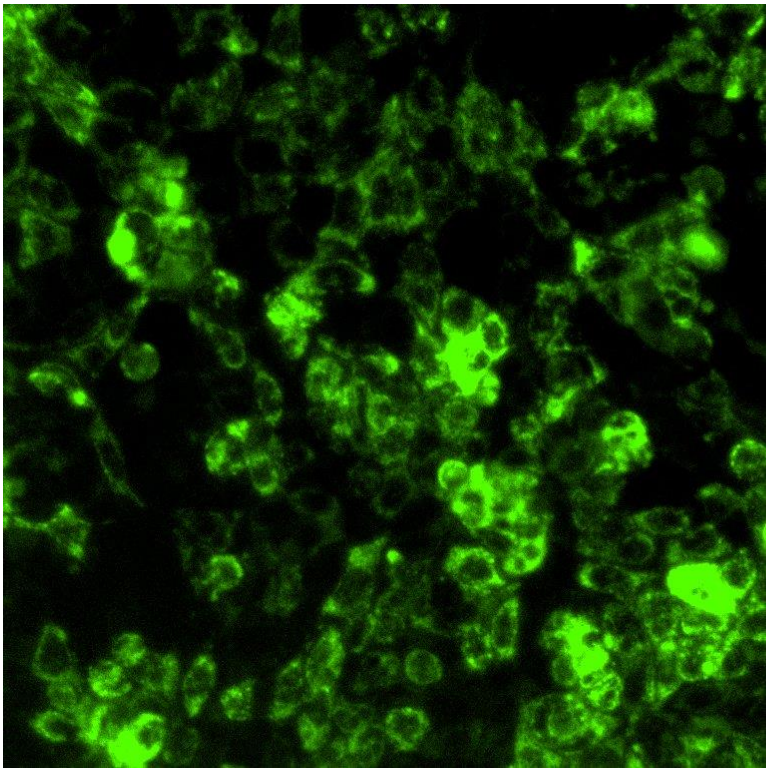

Caco-2, 3×10^4 cells/well, no oil, 24 h (hypoxia dye)

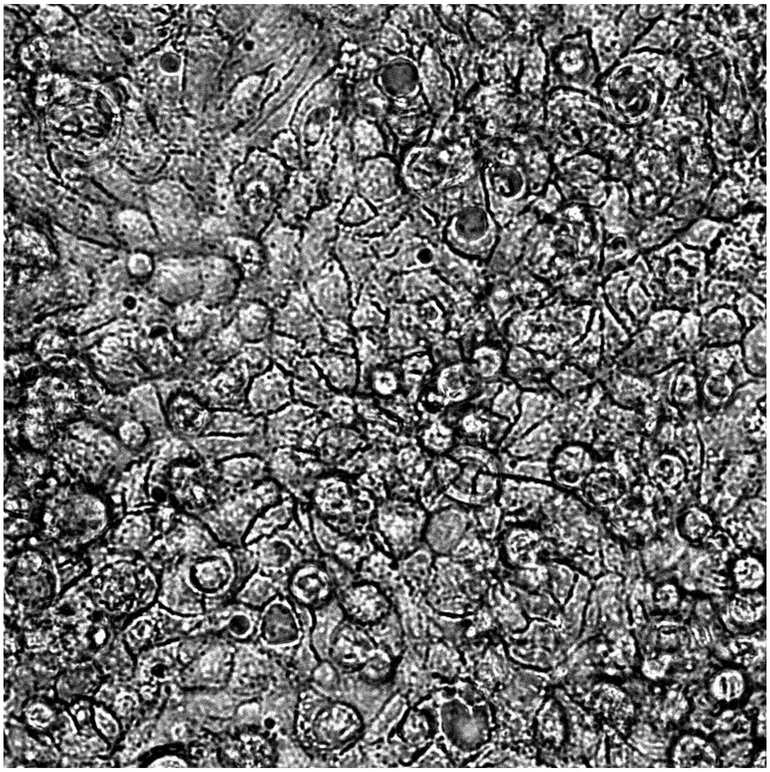

Caco-2, 3×10^4 cells/well, media-2 mm, silicone oil-5 mm-5 cSt, 24 h (bright field)

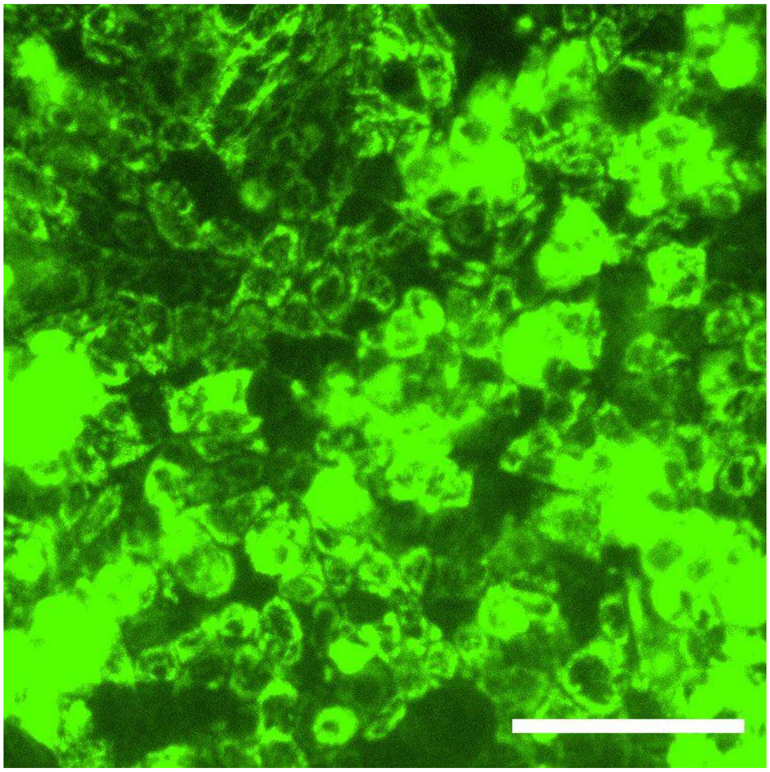

Caco-2, 3×10^4 cells/well, media-2 mm, silicone oil-5 mm-5 cSt, 24 h (hypoxia dye)

Figure S7. Large images showing the typical cell morphologies in Fig. 4a. Scale bar, 100 μm.

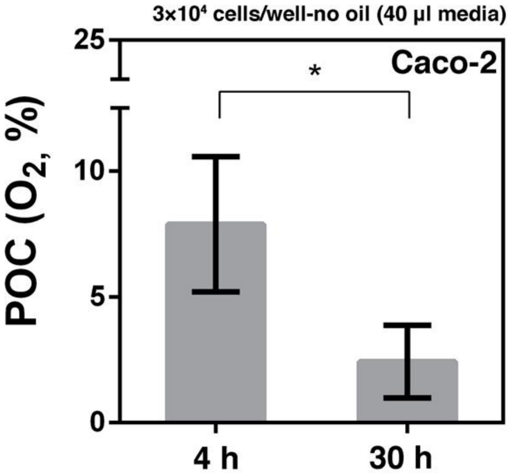

Figure S8. POC of Caco-2 from the condition of large media volume (40 μl/well on a 384-well plate for 4 mm in media depth) without oil overlay. Error bars, mean ± s.d. *$P \leq 0.05$.

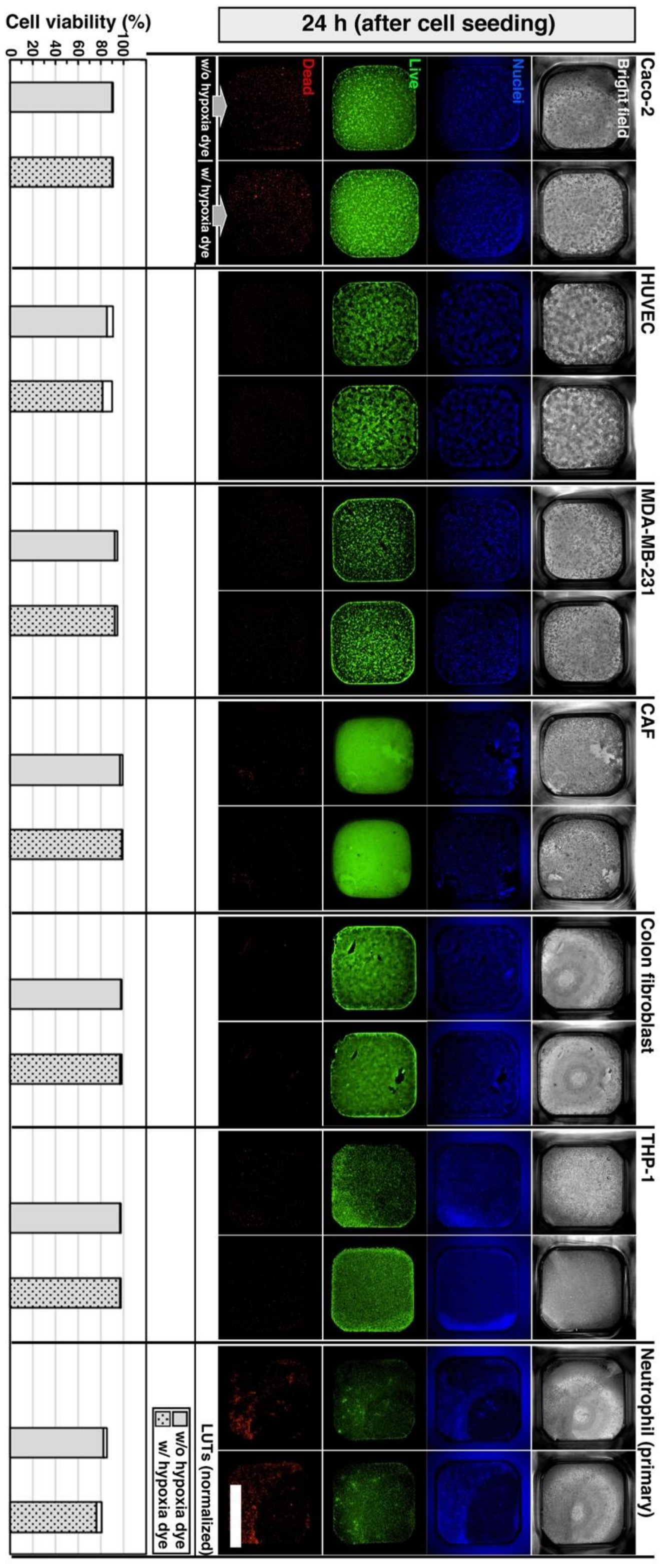

Figure S9. Cell viability of different cell types cultured under oil (24 h after cell seeding with and without hypoxia dye). Each cell type was seeded at 3×10^4 cells/well on a 384-well plate with 20 µl/well of media (for 2 mm in media depth), overlaid with 50 µl of silicone oil (5 cSt) (for an additional 5 mm in oil depth), and cultured up to 24 h for a parallel comparison. The fluorescent images were all processed with normalized LUTs for visualization. Scale bar, 2 mm.

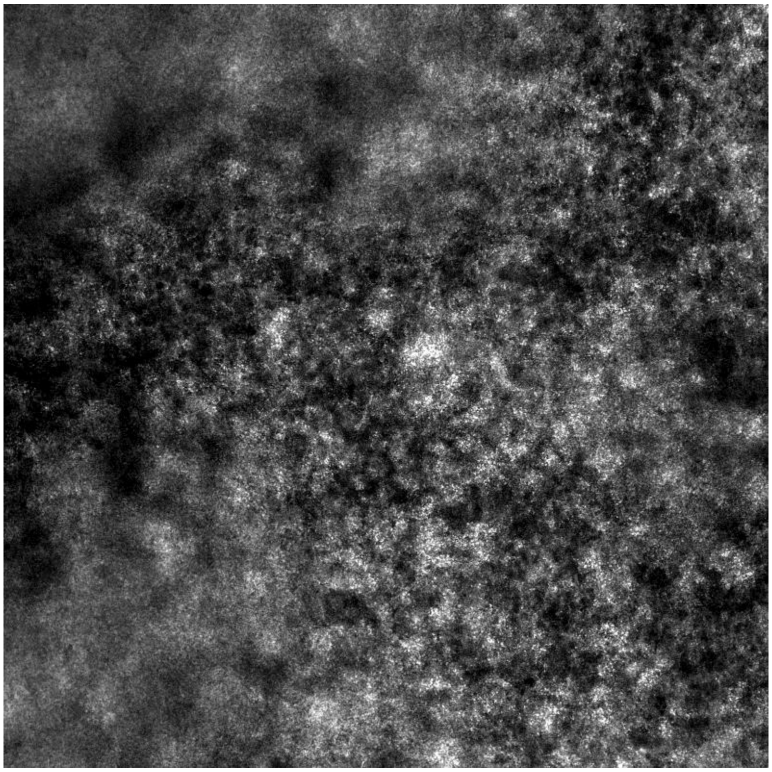

C. albicans, 3×10^4 cells/well, media-2 mm, silicone oil-5 mm-5 cSt, 24 h (bright field)

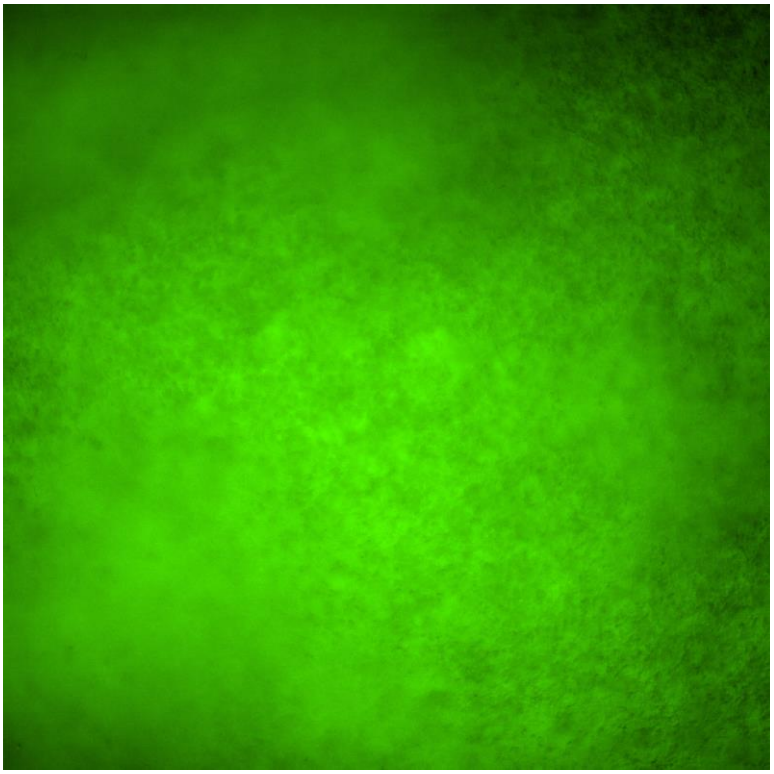

C. albicans, 3×10^4 cells/well, media-2 mm, silicone oil-5 mm-5 cSt, 24 h (hypoxia dye)

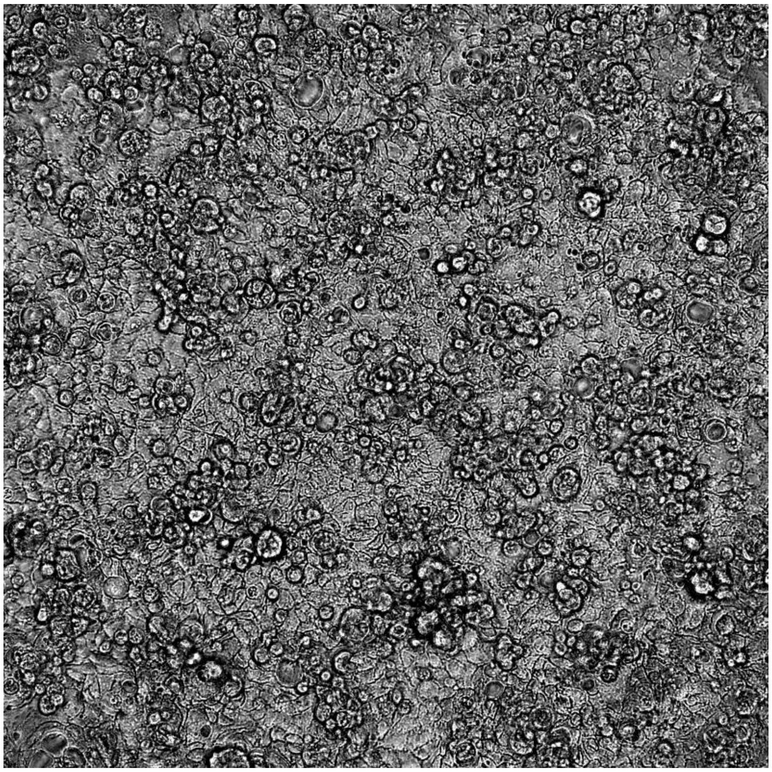

Caco-2, 3×10⁴ cells/well, media-2 mm, silicone oil-5 mm-5 cSt, 24 h (bright field)

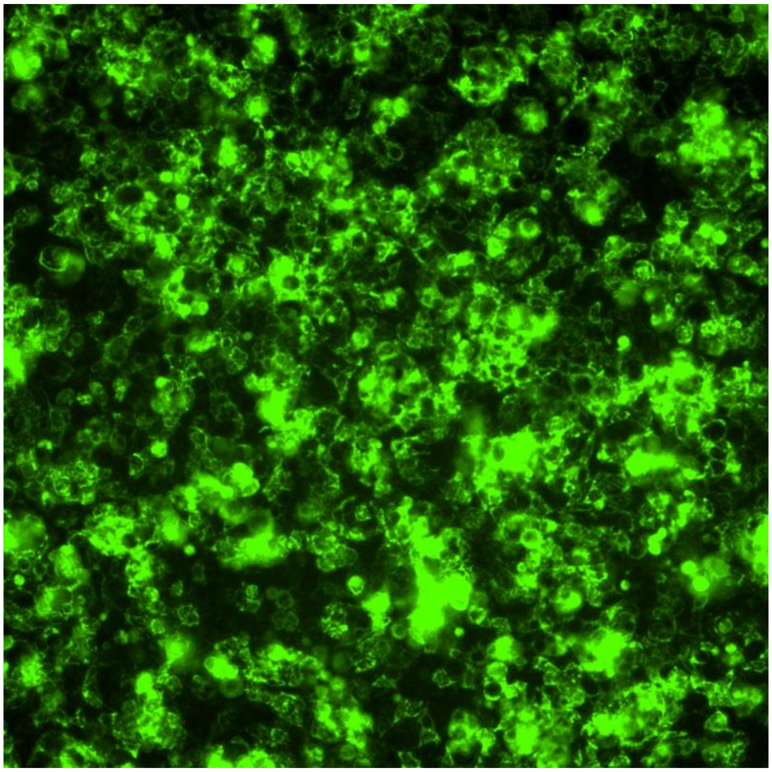

Caco-2, 3×10⁴ cells/well, media-2 mm, silicone oil-5 mm-5 cSt, 24 h (hypoxia dye)

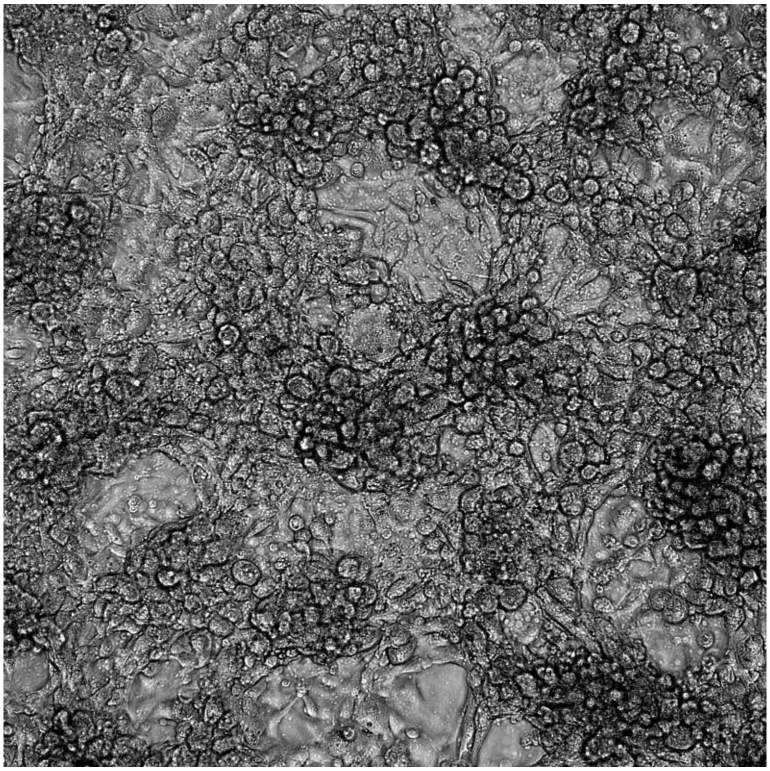
HUVEC, 3×10^4 cells/well, media-2 mm, silicone oil-5 mm-5 cSt, 24 h (bright field)

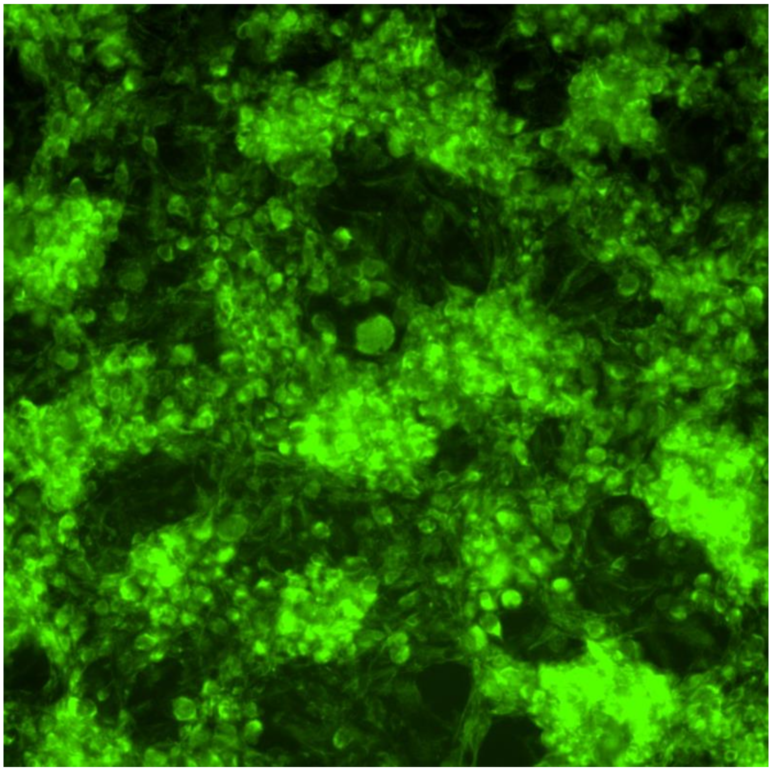
HUVEC, 3×10^4 cells/well, media-2 mm, silicone oil-5 mm-5 cSt, 24 h (hypoxia dye)

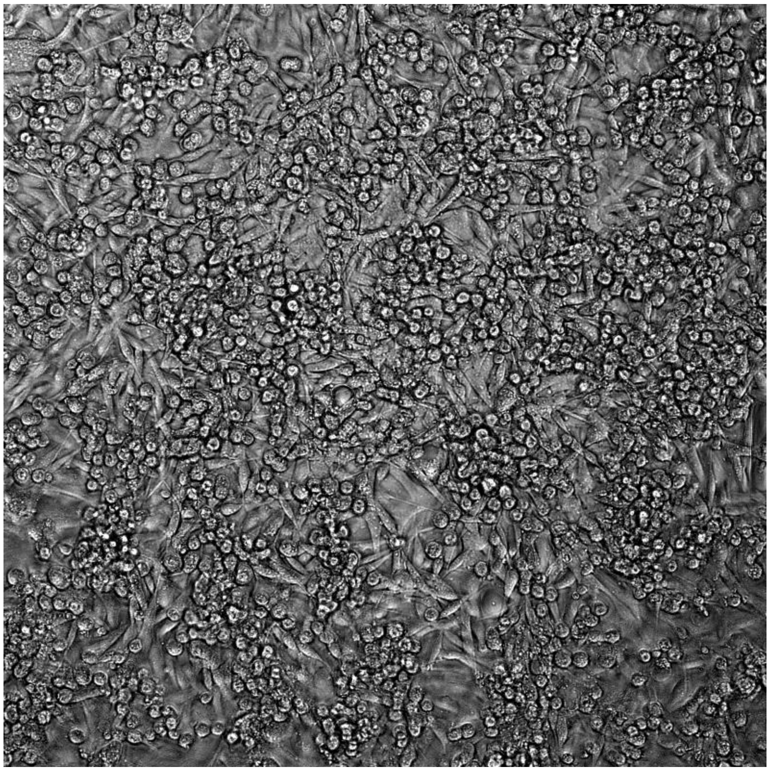

MDA-MB-231, 3×10^4 cells/well, media-2 mm, silicone oil-5 mm-5 cSt, 24 h (bright field)

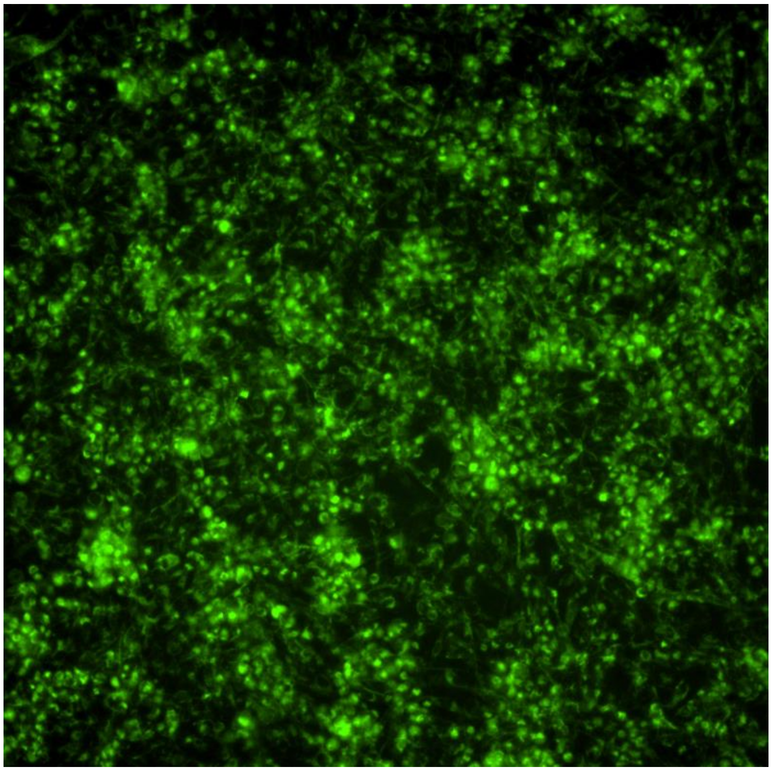

MDA-MB-231, 3×10^4 cells/well, media-2 mm, silicone oil-5 mm-5 cSt, 24 h (hypoxia dye)

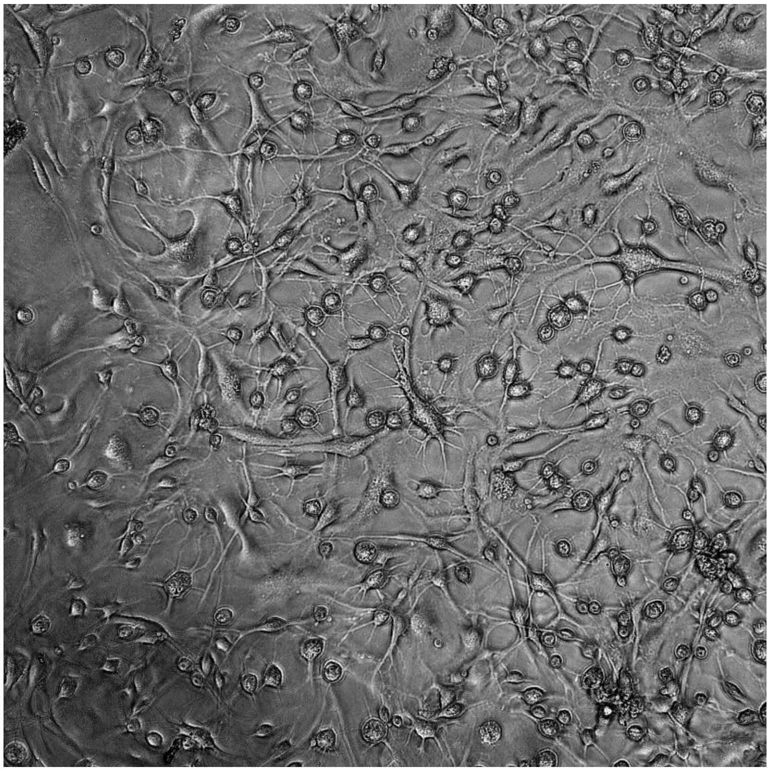

CAF, 3×10^4 cells/well, media-2 mm, silicone oil-5 mm-5 cSt, 24 h (bright field)

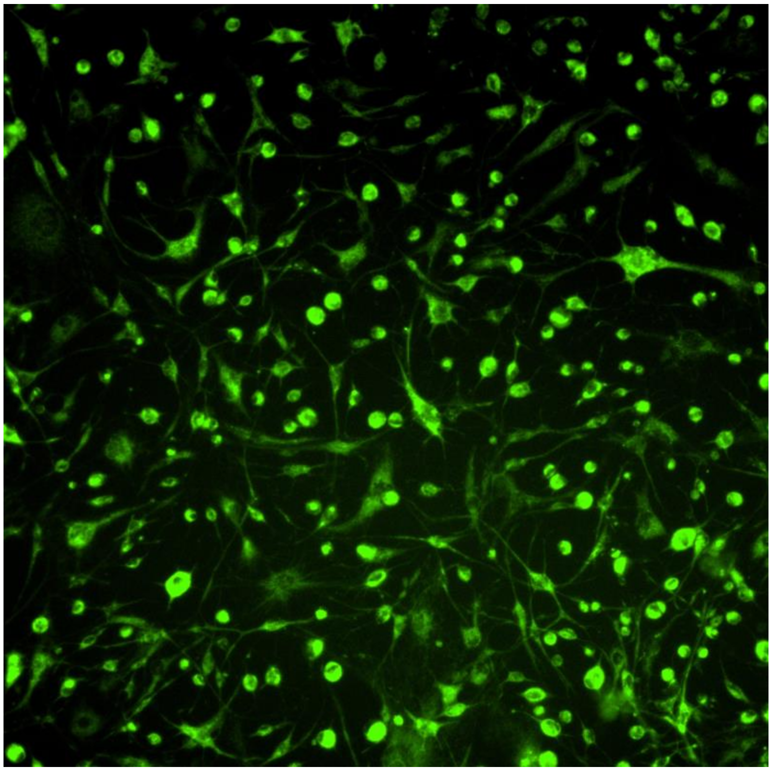

CAF, 3×10^4 cells/well, media-2 mm, silicone oil-5 mm-5 cSt, 24 h (hypoxia dye)

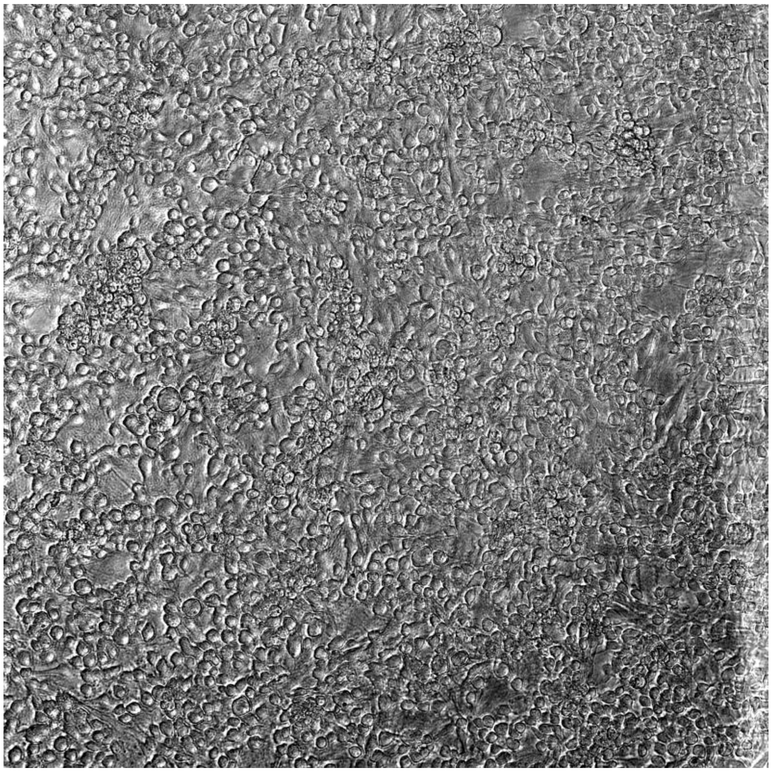
Colon fibroblast, 3×10^4 cells/well, media-2 mm, silicone oil-5 mm-5 cSt, 24 h (bright field)

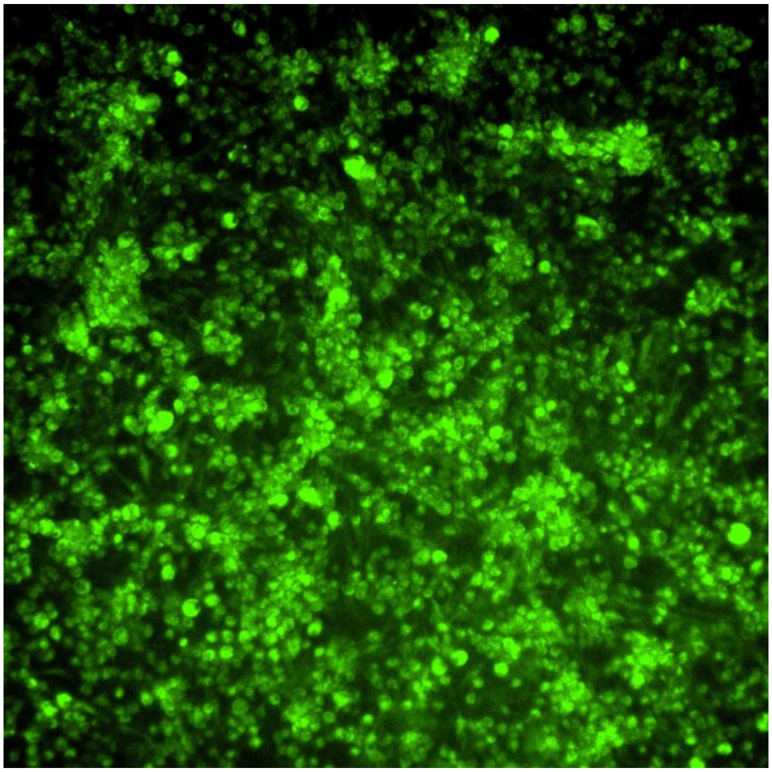
Colon fibroblast, 3×10^4 cells/well, media-2 mm, silicone oil-5 mm-5 cSt, 24 h (hypoxia dye)

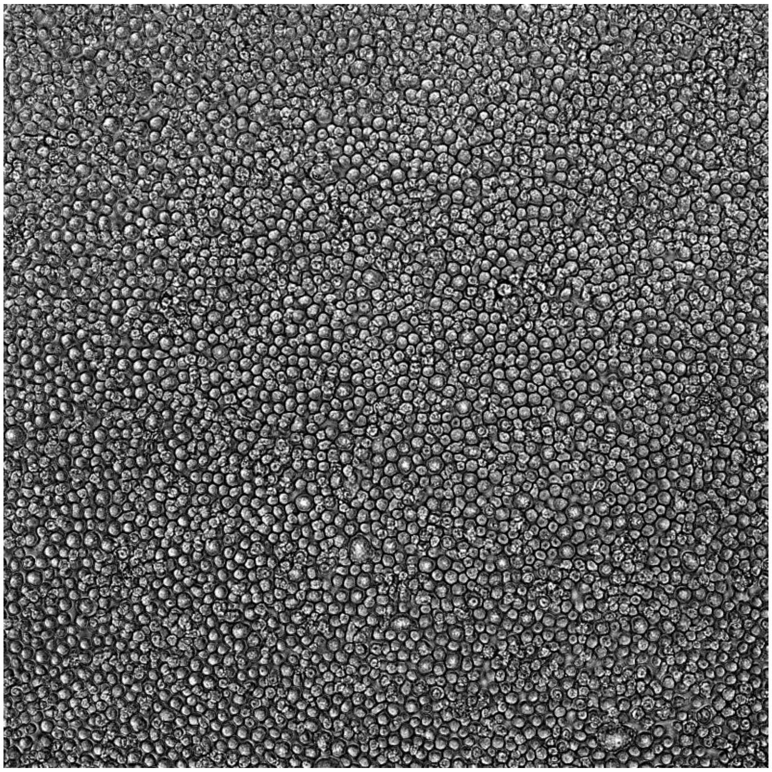
THP-1, 3×10^4 cells/well, media-2 mm, silicone oil-5 mm-5 cSt, 24 h (bright field)

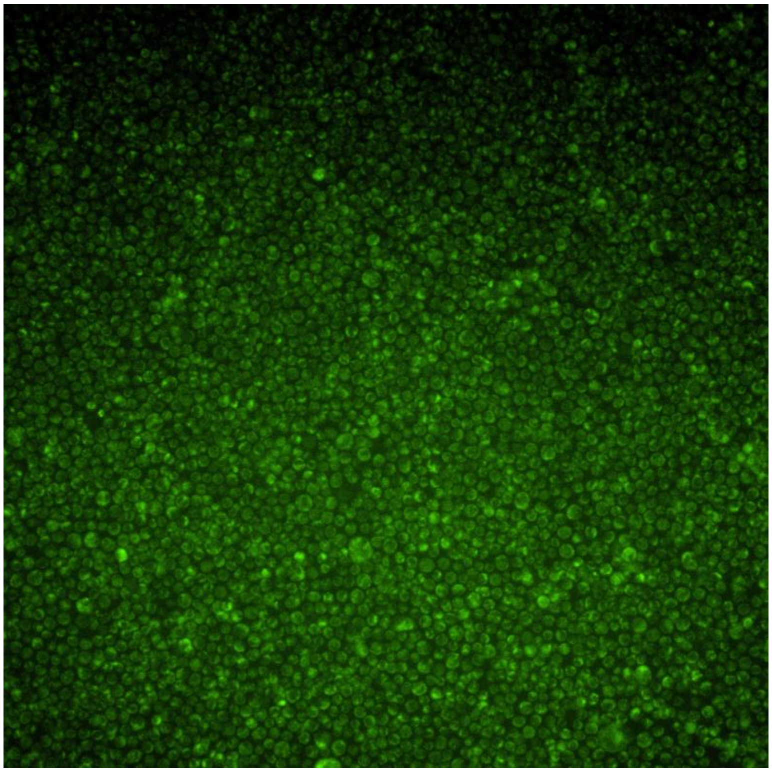
THP-1, 3×10^4 cells/well, media-2 mm, silicone oil-5 mm-5 cSt, 24 h (hypoxia dye)

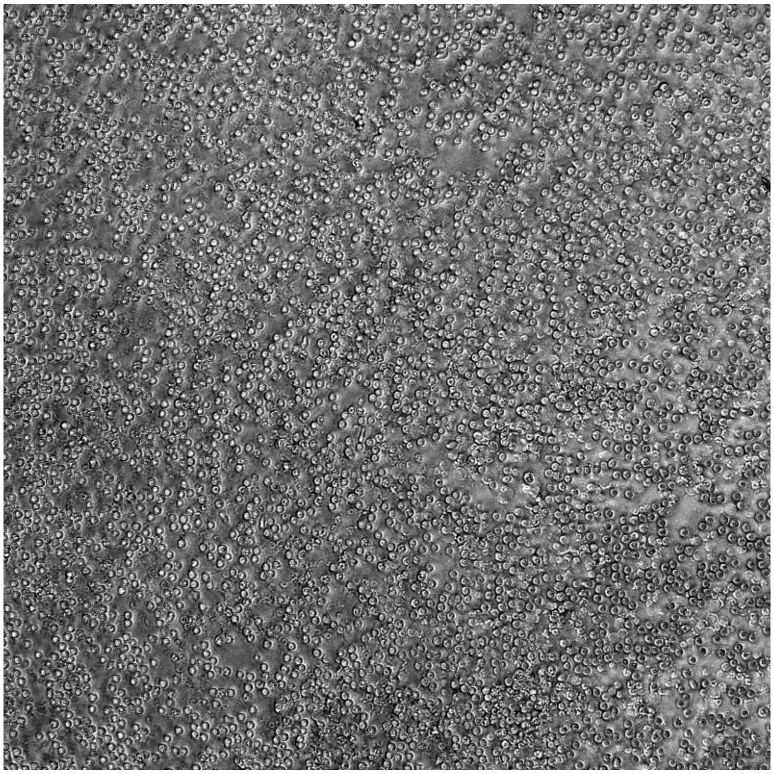

Neutrophil, 3×10^4 cells/well, media-2 mm, silicone oil-5 mm-5 cSt, 24 h (bright field)

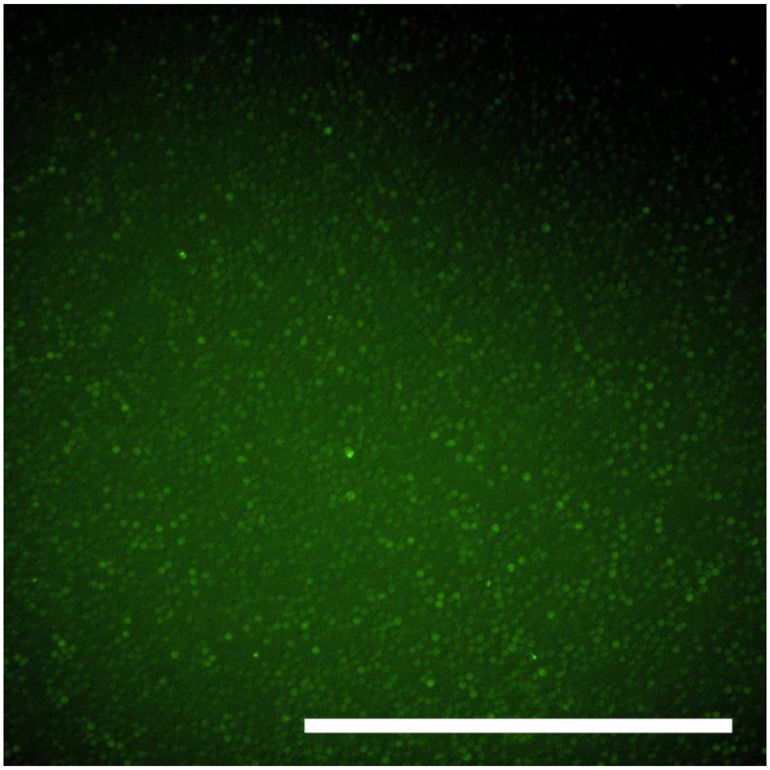

Neutrophil, 3×10^4 cells/well, media-2 mm, silicone oil-5 mm-5 cSt, 24 h (hypoxia dye)

Figure S10. Large images showing the typical cell morphologies in Fig. 5b. Scale bar, 500 μm.

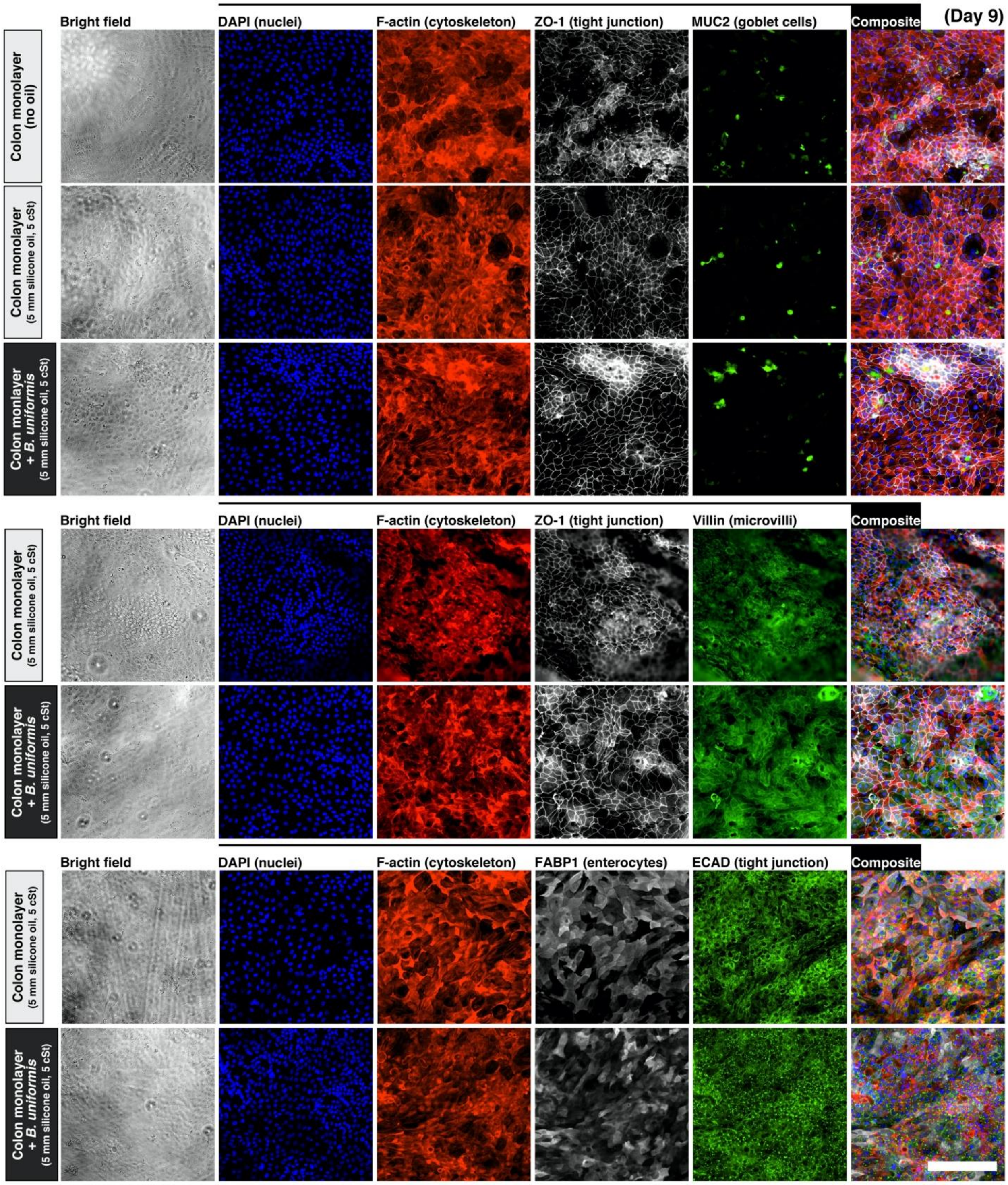

Figure S11. IFS images of primary colon epithelium from monoculture (no-bacteria control) and co-culture with *B. uniformis* under oil on Day 9 (i.e., 24 h after inoculation of the bacteria). The fluorescent images were all processed with normalized LUTs for visualization. Scale bar, 200 μm.

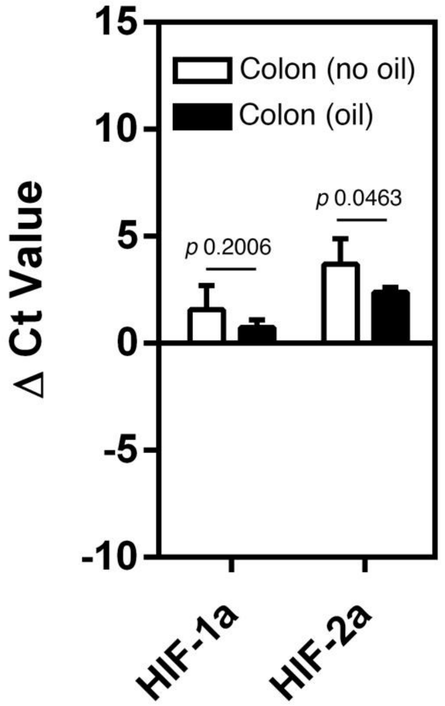

Figure S12. RT-qPCR (ΔCt) results for comparison of HIF-1α and HIF-2α gene expression (Table S2) of the colon epithelium. Lower ΔCt values indicate higher gene expression. The POC of the no-oil controls is 17-19% O_2, and 2-8% from the under-oil culture (Figure 6c). Data were pooled and averaged with 3 replicates of each condition. Error bars, mean ± s.d. The *P* values are shown on the plot.

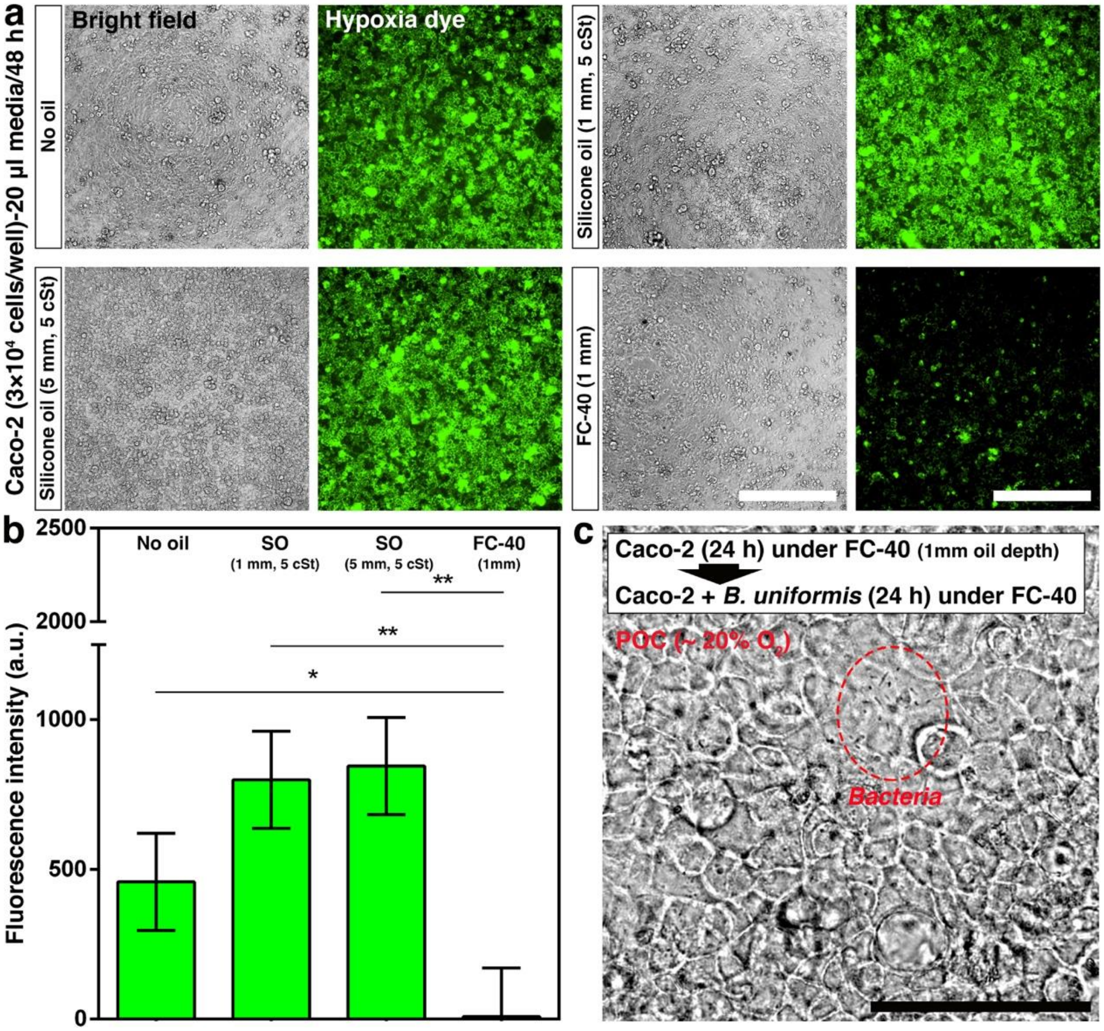

Figure S13. Comparison of hypoxia generation between silicone oil (5 cSt) and fluorinated oil (Fluorinert FC-40). a) Microscopic images (bright field, left; fluorescent, right) of Caco-2 monolayers [3×10^4 cells/well, 20 µl/well of media (for 2 mm media depth), 10 or 50 µl/well silicone oil (5 cSt) (for 1 or 5 mm oil depth, respectively) overlay, 10 µl/well fluorinated oil (FC-40) (for 1 mm in oil depth)] cultured on a 384-well plate for 48 h. The fluorescent images of hypoxia dye were processed with parallel LUTs. Scale bars, 500 µm. b) IOC (fluorescence intensity of hypoxia dye) of each condition. Error bars, mean ± s.d. $^*P \leq 0.05$, and $^{**}P \leq 0.01$. c) Co-culture of Caco-2 monolayer from (a) with *B. uniformis* [inoculum density, $OD_{600} = 0.1$, 1:20 v/v ratio (1 µl bacteria:20 µl media)] under fluorinated oil (FC-40, 1 mm oil depth). POC was measured for about 20% O_2 with the fluorinated oil overlay. The bacteria (the red dashed line circle) showed little growth after 24 h co-culture under FC-40. Scale bar, 200 µm.

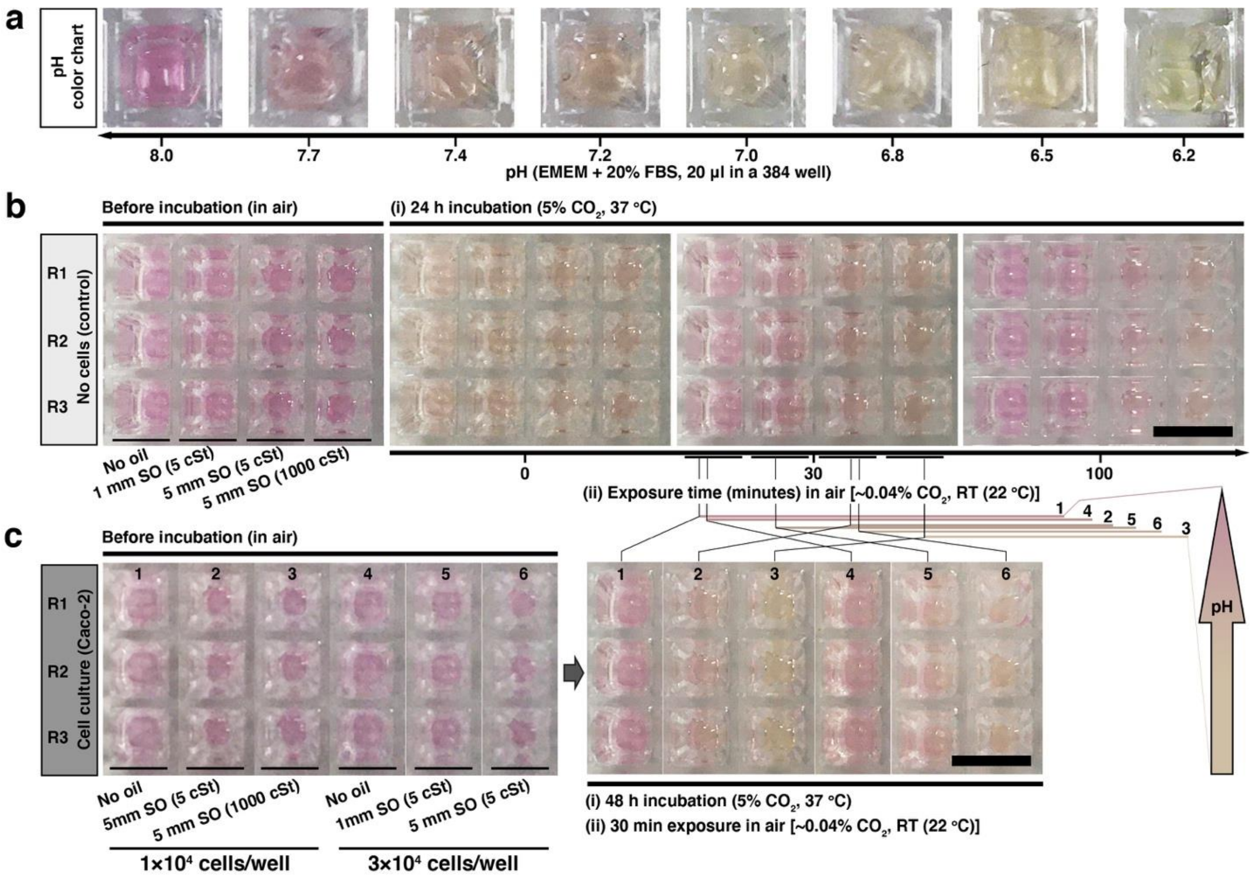

Figure S14. Colorimetric analysis of pH of the culture media before/after incubation, and after exposure in air. a) A pH color chart of phenol red (30 µM in EMEM + 20% FBS, 20 µl/well on a 384-well plate). b) The control of a no-cell plate with different oil [silicone oil (SO)] overlays. c) The Caco-2 plate [1×10^4 or 3×10^4 cells/well, 20 µl/well of media (for 2 mm in media depth)] with different oil overlays. CO_2 dissolved in the culture media diffused out through the oil layer over time, which led to different recovery rates of pH from acidic to basic across the tested conditions. The oil overlay stabilized the pH in culture media during device transfer or operation in an atmospheric ambient environment. Scale bars, 4 mm.

Table S1. Compiled information of cell types and culture media.

**Cell type (tissue origin)**	**Name**	**Culture media**
Endothelium (blood vessel)	HUVEC (human umbilical vein endothelial cell)	Endothelial basal medium-2 (EBM-2) (Lonza, 0019086) + 10% fetal bovine serum (FBS) (Thermo Fisher Scientific, 10437010) + 1% Penicillin-Streptomycin (Pen-Strep) (Thermo Fisher Scientific, 15070063)
Epithelium (colon cancer)	Caco-2 (cancer coli-2)	Eagle's minimal essential medium (EMEM) (Sigma Aldrich, M4655) + 20% FBS + 1% Pen-Strep
Epithelium (breast cancer)	MDA-MB-231	Dulbecco's Modified Eagle's medium (DMEM) (Thermo Fisher Scientific, 11960051) + 10% FBS + 1% Pen-Strep
Fibroblast (normal)	Colon fibroblasts	Fibroblast media (ScienCell, C2301), 384-well plates coated with gelatin solution (Thermo Fisher Scientific, S25335) at 37 ^{o}C for 15 min and then aspirated.
Fibroblast (tumor-associated)	CAF (cancer-associated fibroblasts) (breast)	DMEM + 10% FBS + 1% Pen-Strep
Blood cells (monocytes)	THP-1	Roswell Park Memorial Institute (RPMI) 1640 (Thermo Fisher Scientific, 11875085I) + 10% FBS + 1% Pen-Strep + 1% lactose
Blood cells (neutrophils)	Isolated from whole blood	EBM-2 + 5% FBS + 1% Pen-Strep
Primary epithelium (colon)	Colon organoids	Intestinal stem cell media [45% L-WRN conditioned media,[1] 45% midgut media,[2] 10% FBS, 50 ng/ml epidermal growth factor (EGF), 500 nM A-83-01, 10 μM SB202190, 10 nM [Leu^{15}]-Gastrin-1, 1 mM N-Acetylcysteine, 10 μM Y-27632, 2.5 μM CHIR99021, 2.5 μM Thiazovivin, and 100 μg/ml Primocin]
	Colon monolayer	Intestinal stem cell media (see above)<br><br>Differentiation media (5% L-WRN conditioned media, 85% midgut media, 10% FBS, 50 ng/ml EGF, 500 nM A-83-01, 10 nM [Leu^{15}]-Gastrin-1, 1 mM N-Acetylcysteine)
Fungi	*Candida albicans* (*C. albicans*, CMM 16 PES1 mutant)	RPMI 1640
Bacteria	mCherry-labelled *Bacteroides uniformis* (*B. uniformis*, DMS 6597)	Anaerobe Basal Broth (Oxoid, CM0957)<br>Brain Heart Infusion Broth (Sigma Aldrich, 53286) (for conjugation)

Table S2: The panel of genes in RT-qPCR and related protein function.

**Gene**		**Protein function**	**Source**
Proliferation	MKI67	Cell proliferation marker	Thermo Fisher Scientific, Hs04260396_g1
Differentiation	Axis inhibition protein 2 (Axin2)	A surrogate marker of intestinal stem cell activity (targeting Wnt signaling pathway)	Thermo Fisher Scientific, Hs00610344_m1
	Trefoil factor 1 (TFF1)	An enterocyte marker (stabilization of mucus layer, healing of the epithelium)	Thermo Fisher Scientific, Hs00907239_m1
	Sucrase-isomaltase (SI)	An enterocyte marker (digestion of dietary carbohydrates)	Thermo Fisher Scientific, Hs00356112_m1
	Villin	Microvilli marker	Thermo Fisher Scientific, Hs01031739_m1
	Mucin 2 (MUC2)	Goblet cell marker (epithelial lining)	Thermo Fisher Scientific, Hs03005103_g1
Hypoxia response	Hypoxia-inducible factor (HIF-1α/HIF-2α)	Transcription factors (developmental response to hypoxia)	Thermo Fisher Scientific, Hs00153153_m1 (HIF-1α), Hs01026149_m1 (HIF-2α)
Housekeeping (Reference genes)	GAPDH	N/A	Thermo Fisher Scientific, Hs01922876_m1
	HPRT	N/A	Thermo Fisher Scientific, Hs02800695_m1
	RPLP0	N/A	Thermo Fisher Scientific, Hs99999902_m1

Supplementary References

[1] K. L. VanDussen, N. M. Sonnek, T. S. Stappenbeck, Stem Cell Res. 2019, 37, 101430.

[2] M. M. Mahe, N. Sundaram, C. L. Watson, N. F. Shroyer, M. A. Helmrath, J. Vis. Exp. 2015, DOI 10.3791/52483.

SI Section – COMSOL Tutorial

1. Install COMSOL Multiphysics 5.6 on a PC. Double-click the icon on desktop.

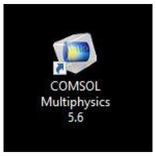

You will see the welcome page as shown below:

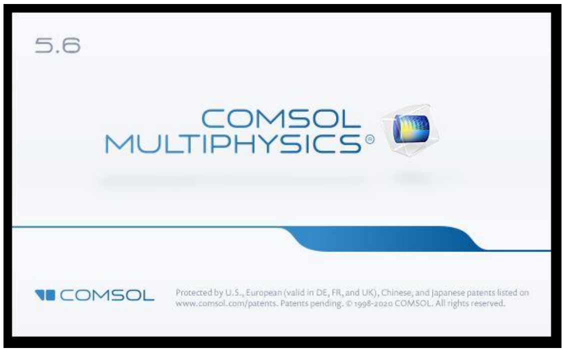

2. File → Licensed and Used Products

Make sure the Chemical Reaction Engineering Module is installed and selected.

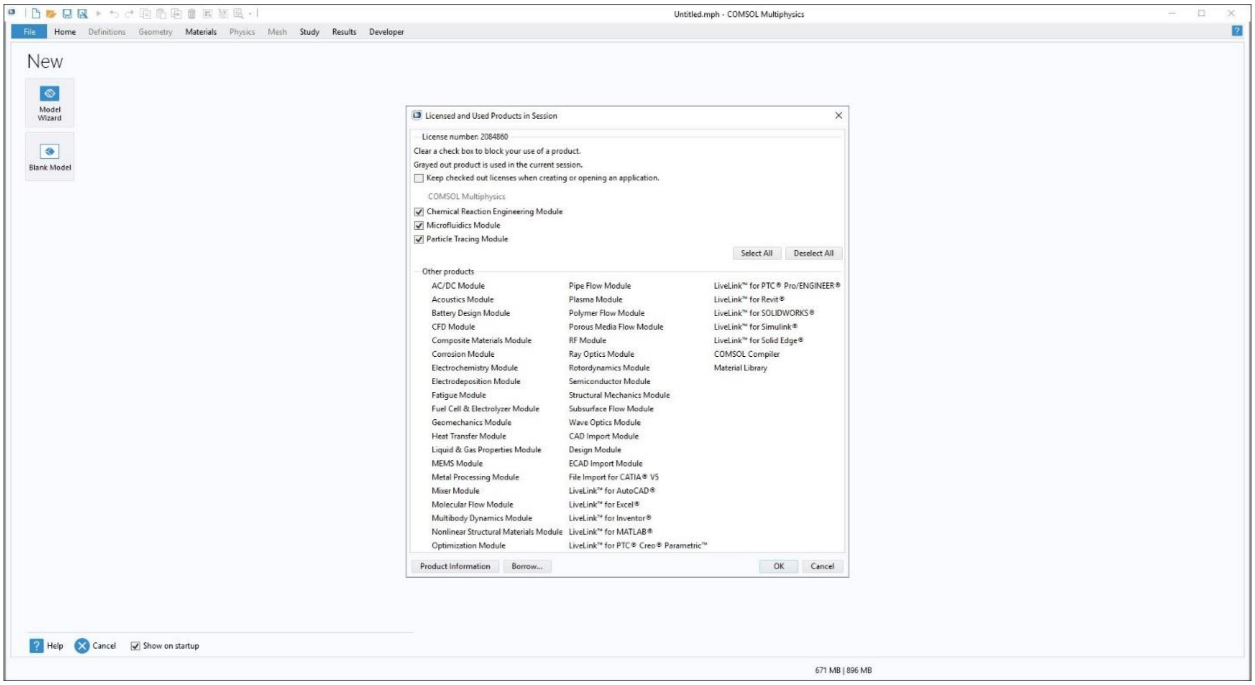

3. File → Open → Select an .mph file

For example

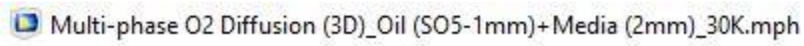

When an .mph file is opened, you will see the COMSOL UI as shown below:

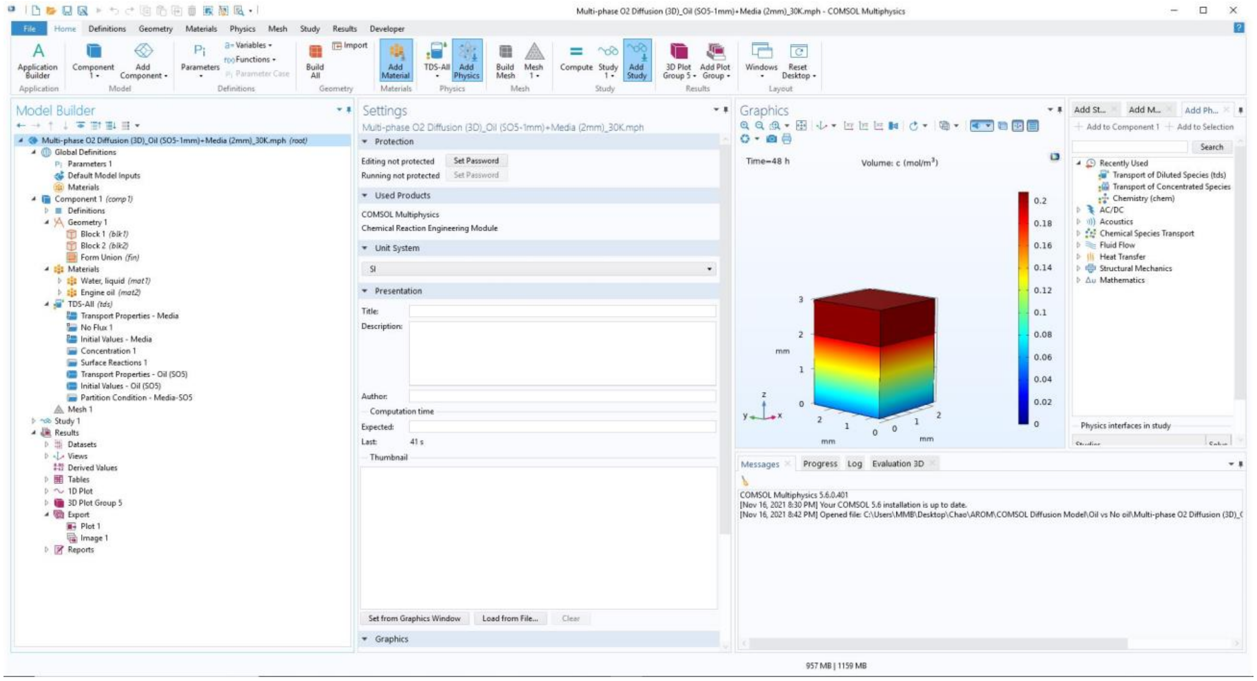

4. Global Definitions → Parameter 1
You will see the Parameters in the Settings (as highlighted by the red box):

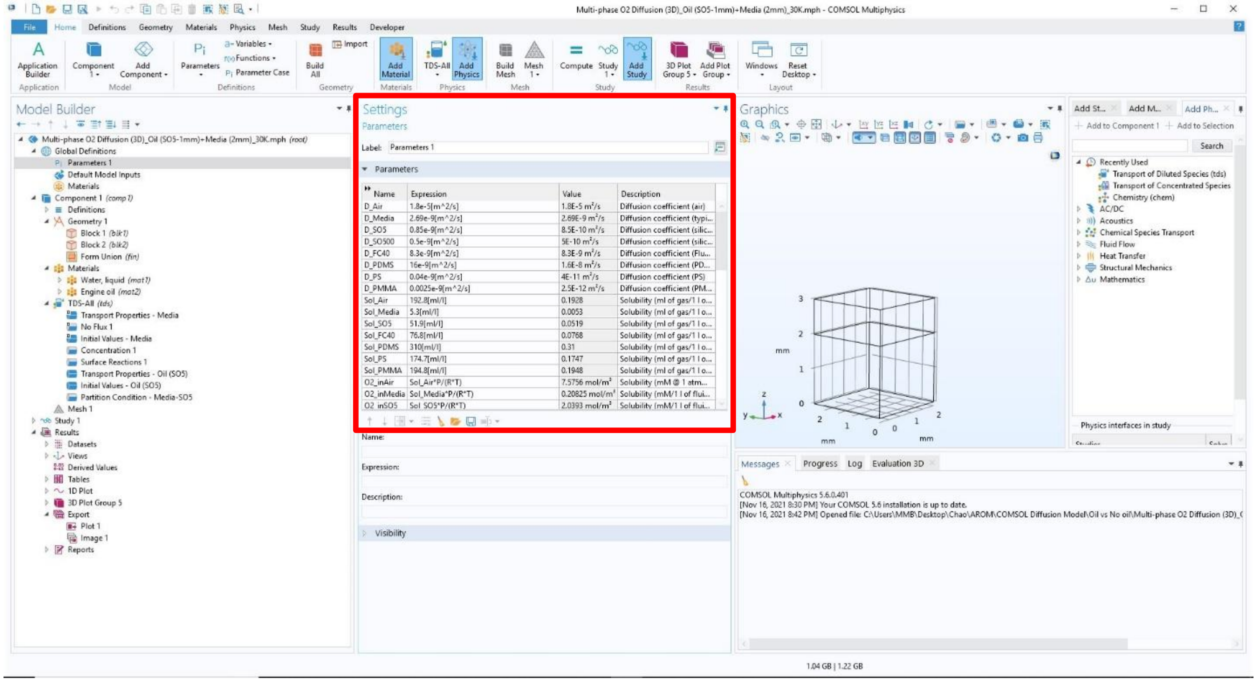

5. Global Definitions → Default Model Inputs
You will see the Default Model Inputs in the Settings (as highlighted by the red box):

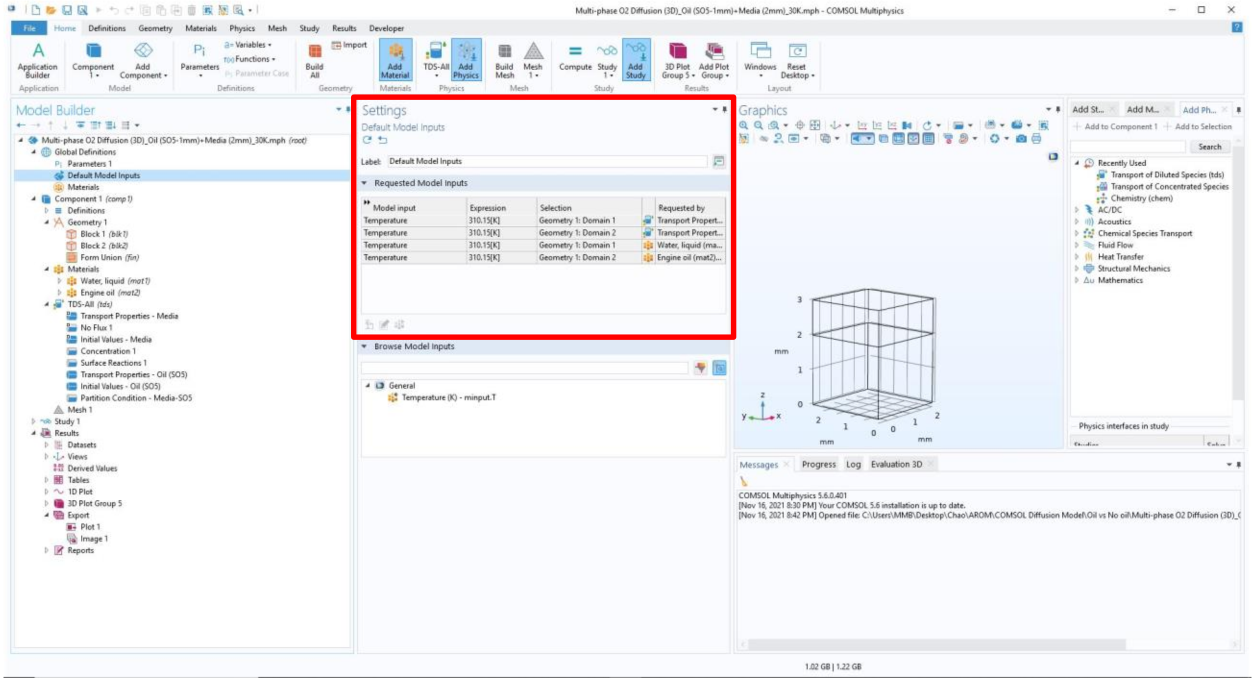

Temperature can be changed by putting a different number in Expression.

6. Component 1 → Geometry 1 → Block 1, Block 2, …
You will see the Block in the Settings (as highlighted by the red box):

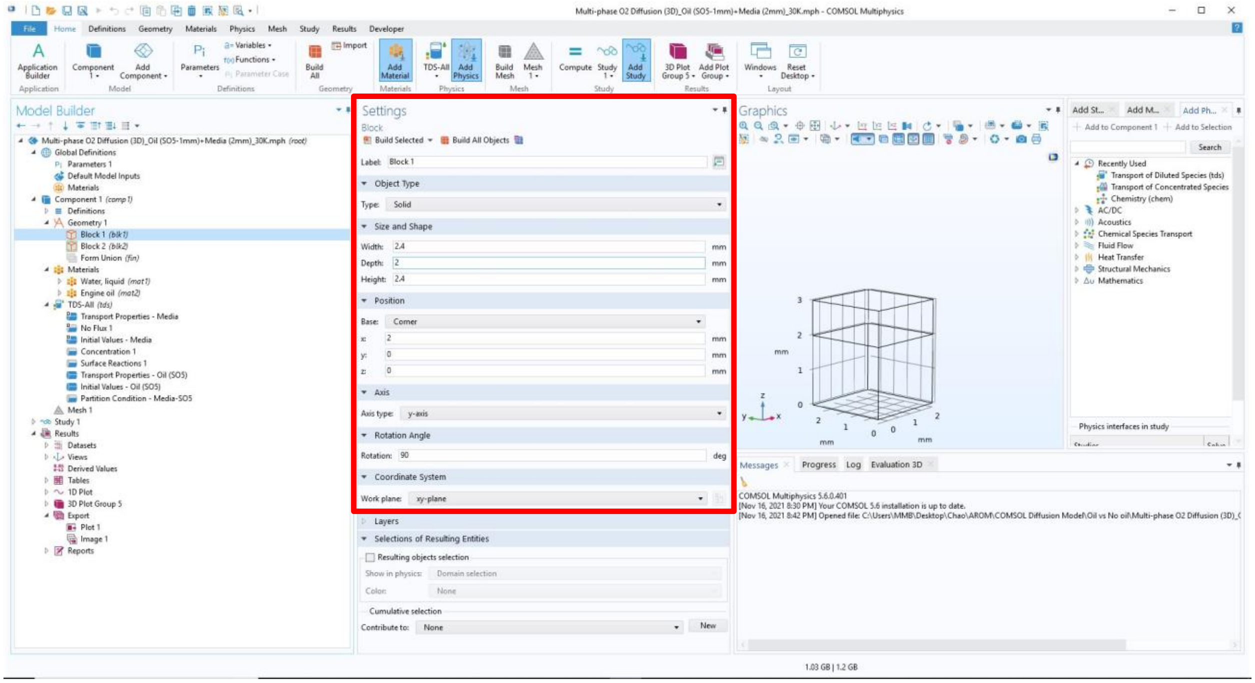

There you can define the object type, size and shape, position, and orientation based on the design in an experiment.

7. Materials
You will see the Material in the Settings (as highlighted by the red box):

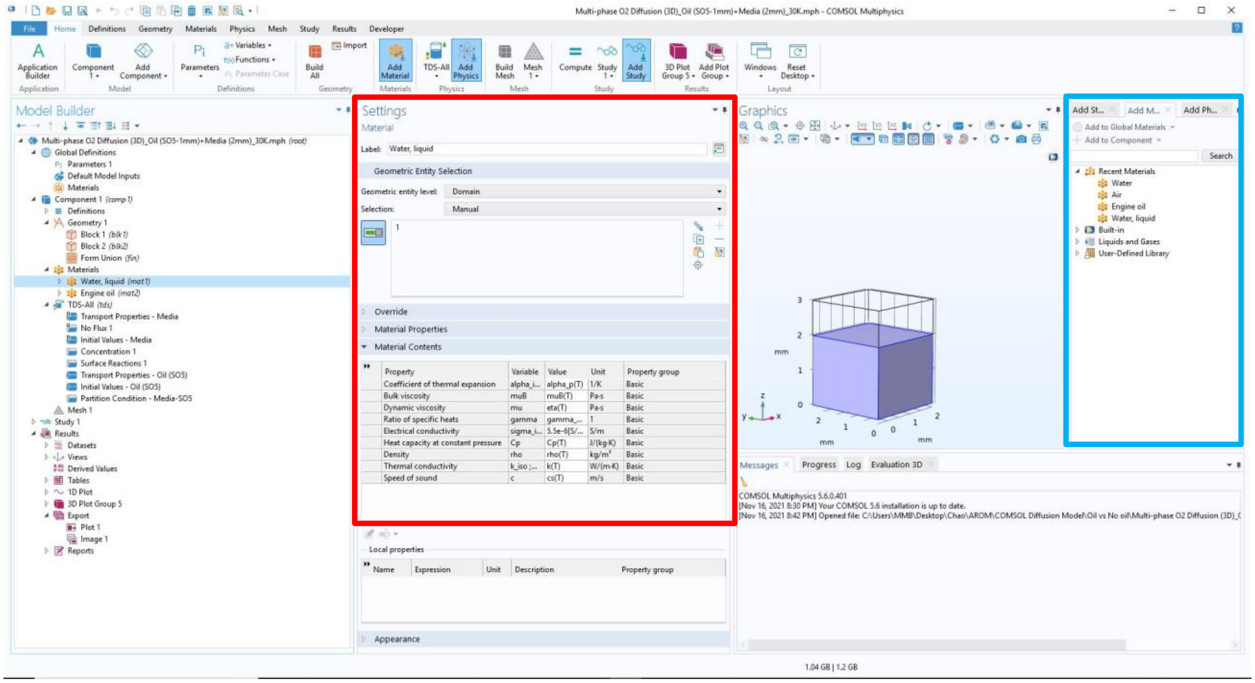

You can add new materials using “Add Material” (as highlighted by the blue box) from the database.

8. Physics (Transport of Diluted Species (*tds*) in the demo)

You will see the components of physics that define the system (as highlighted by the red box):

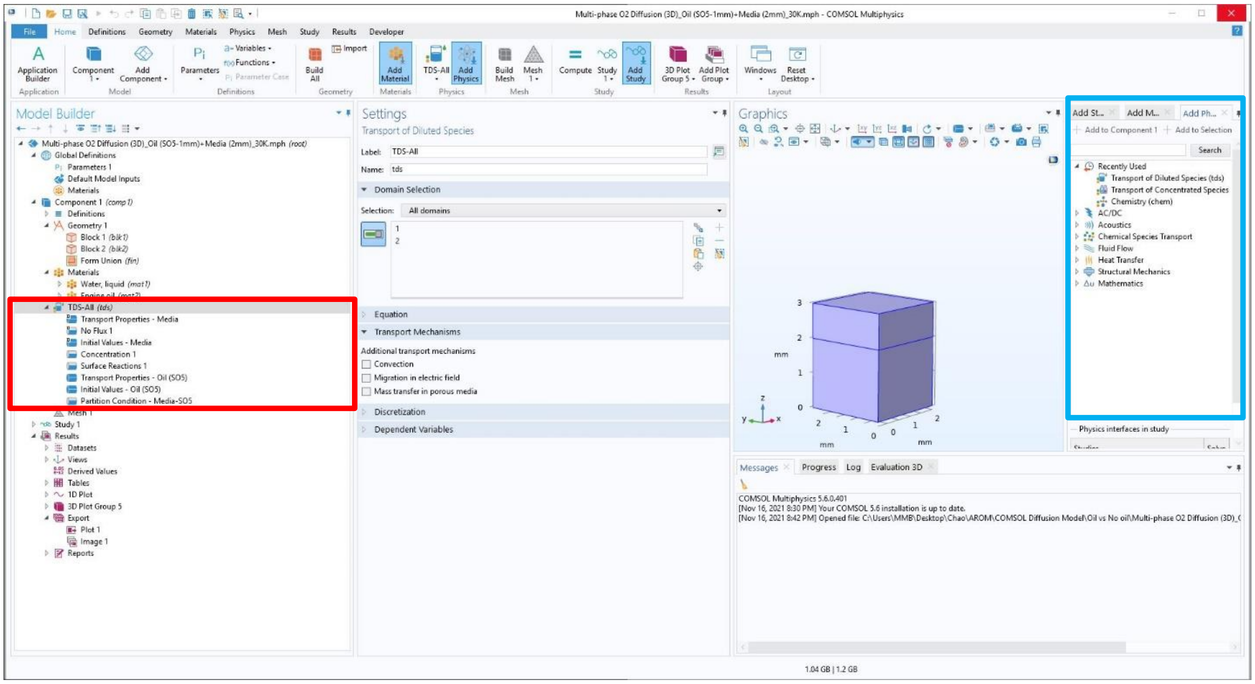

You can switch to other physics modules using “Add Physics” (as highlighted by the blue box) from the database.

9. Mesh

You will see Mesh in the Settings (as highlighted by the red box):

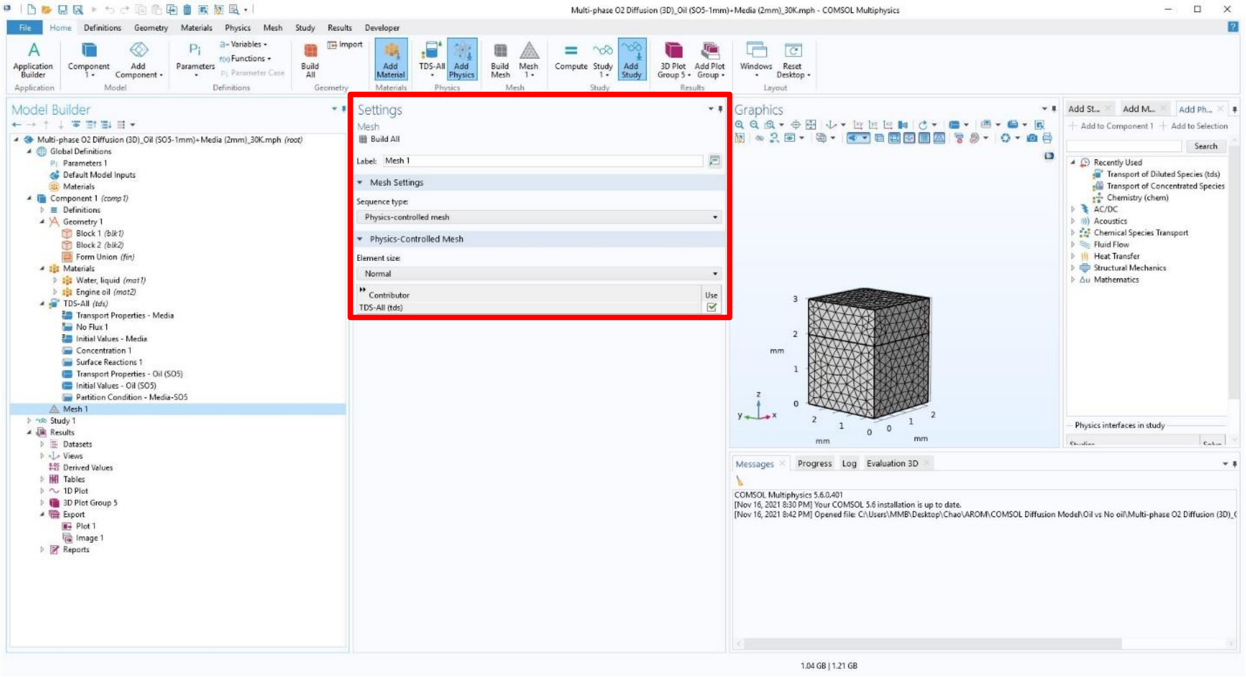

The default element size is Normal. You can use coarser (faster) or finer (slower) mesh as needed.

10. Study 1 → Step 1: Time Dependent

You will see Time Dependent in the Settings (as highlighted by the red box):

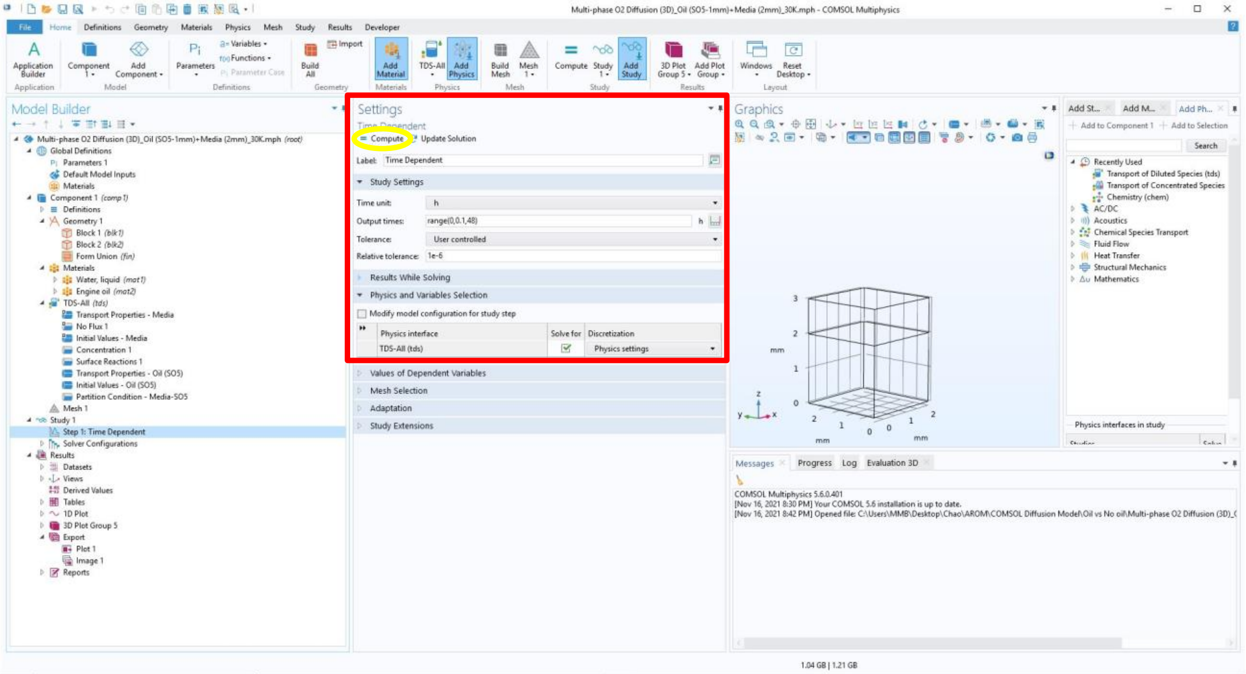

You can select a time unit and the range of output times. The tolerance can be defined as well.

11. Results → 1D Plot → Point Graph 1

You will see Point Graph in the Settings (as highlighted by the red box):

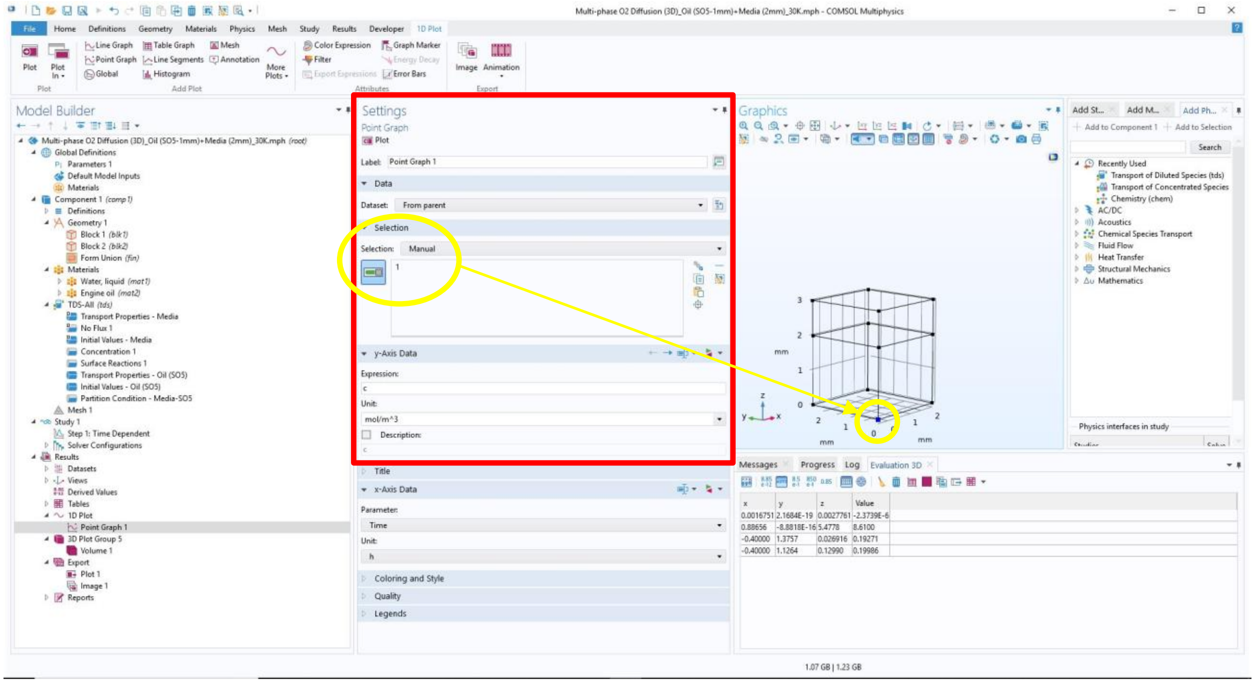

Go to Selection and pick the point of interest for the output (as highlighted by the yellow circles). After everything is set, click “Compute” in Step 10 (as highlighted by the yellow circle).

12. Results → 1D Plot or 3D Plot
Click tabs (1D Plot or 3D Plot) and you will see the 1D and 3D graphs.

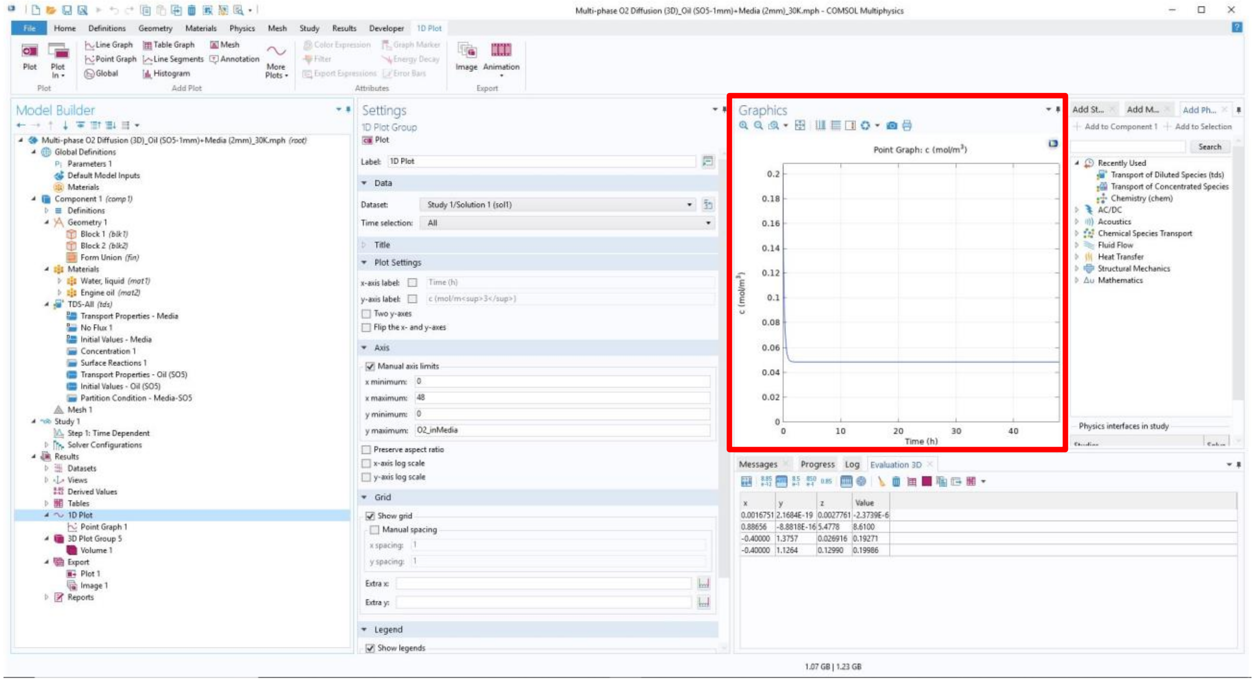

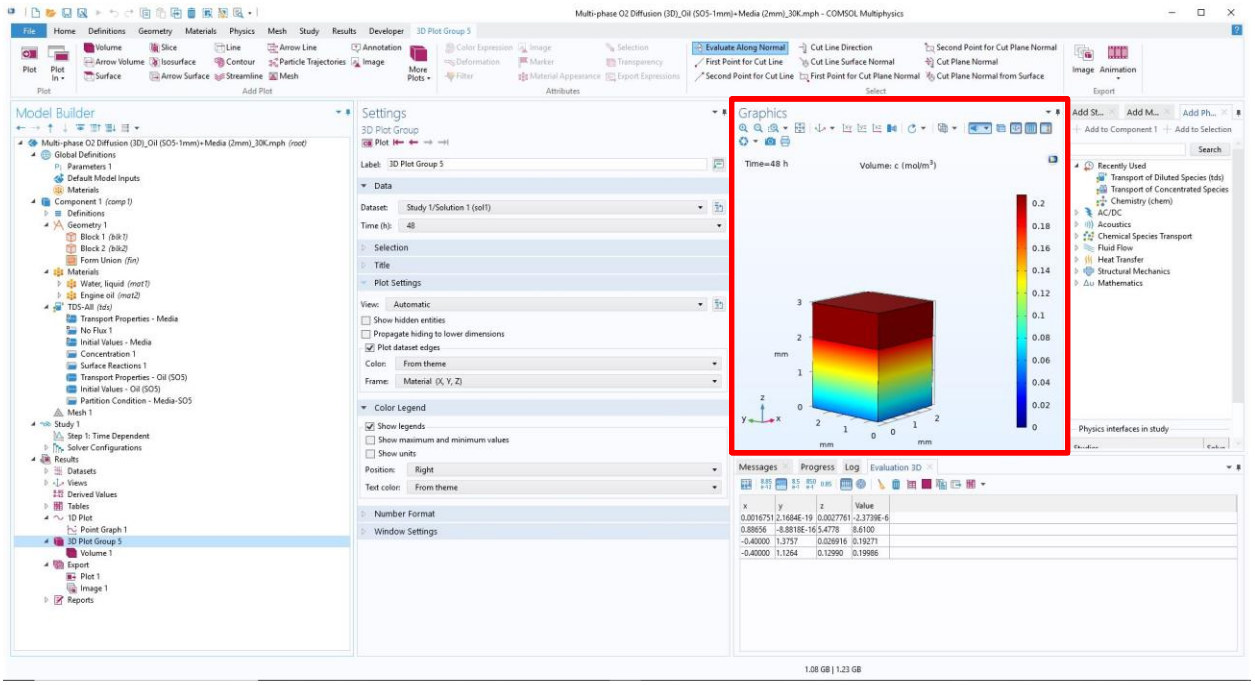

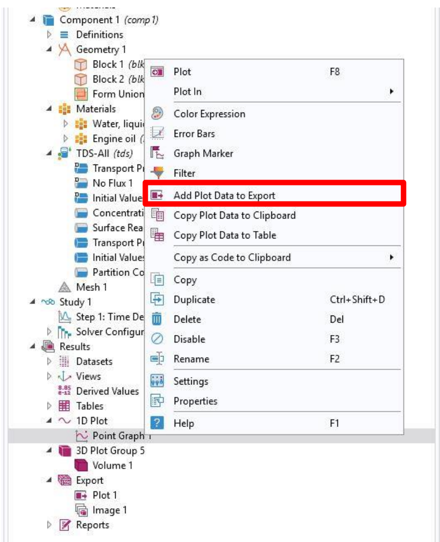

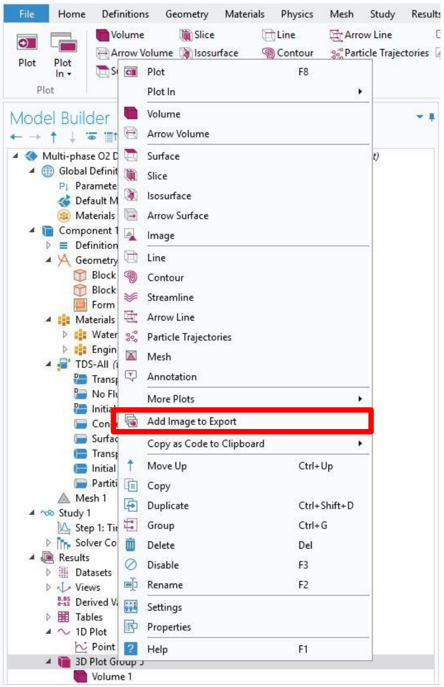

Right click on the tabs (Point Graph 1 or Volume 1) and you can "Add Plot Data to Export". Or Right click on the tabs (1D Plot or 3D Plot) and you can "Add Image to Export".

13. Export → Plot 1 or Image 1

Go to Export. You can save the plots in .txt (i.e., spreadsheet) and the images in a selected format (e.g., TIFF, JPEG, or PNG) (as highlighted by the red boxes).

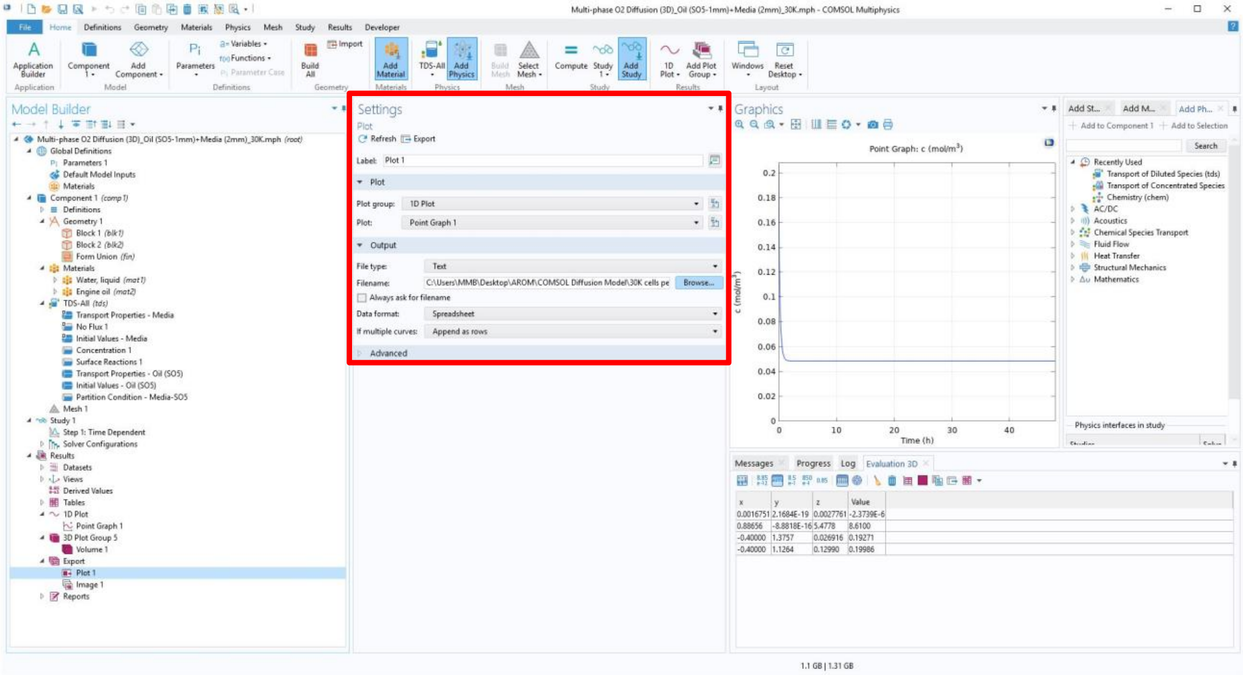
Multi-phase O2 Diffusion (3D)_Oil (SO5-1mm)+Media (2mm)_30K.mph - COMSOL Multiphysics
Settings
Plot
Refresh Export
Label: Plot 1
Plot
Plot group: 1D Plot
Plot: Point Graph 1
Output
File type: Text
Filename: C:\Users\MMB\Desktop\AROM\COMSOL Diffusion Model\30K cells pe
Browse...
Always ask for filename
Data format: Spreadsheet
If multiple curves: Append as rows
Advanced
Graphics
Point Graph: c (mol/m³)
Time (h)
Messages Progress Log Evaluation 3D
1.1 GB | 1.31 GB

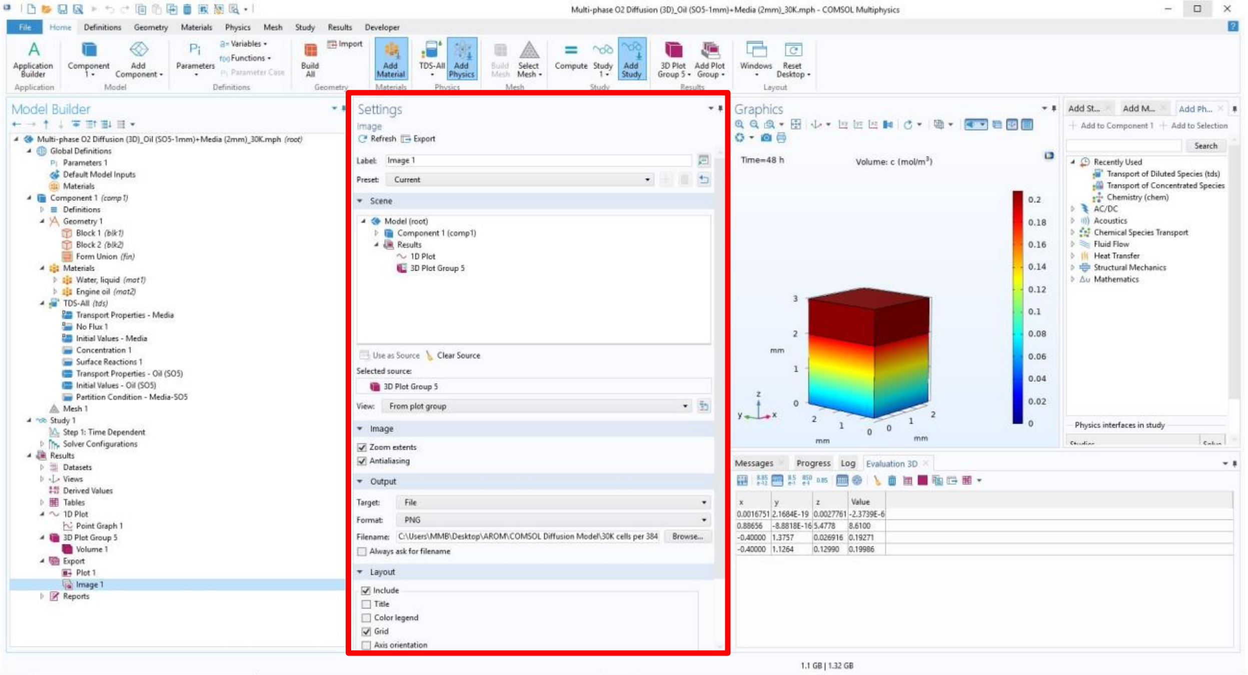
Multi-phase O2 Diffusion (3D)_Oil (SO5-1mm)+Media (2mm)_30K.mph - COMSOL Multiphysics
Settings
Image
Refresh Export
Label: Image 1
Preset: Current
Scene
Model (root)
Component 1 (comp1)
Results
1D Plot
3D Plot Group 5
Use as Source Clear Source
Selected source:
3D Plot Group 5
View: From plot group
Image
Zoom extents
Antialiasing
Output
Target: File
Format: PNG
Filename: C:\Users\MMB\Desktop\AROM\COMSOL Diffusion Model\30K cells per 384
Browse...
Always ask for filename
Layout
Include
Title
Color legend
Grid
Axis orientation
Graphics
Time=48 h
Volume: c (mol/m³)
Messages Progress Log Evaluation 3D
1.1 GB | 1.32 GB
